# MS4A6A/Ms4a6d deficiency disrupts neuroprotective microglia functions and promotes inflammation in Alzheimer’s disease model

**DOI:** 10.1186/s13024-025-00887-0

**Published:** 2025-08-28

**Authors:** Hai-Shan Jiao, Yi-Jun Ge, Liang-Yu Huang, Ying Liu, Bang-Sheng Wu, Piao-Piao Lian, Yi-Ning Hao, Shan-Shan Han, Yi-Ting Li, Kai-Min Wu, Chen-Yun Wu, Tian-Lin Cheng, Peng Yuan, Jin-Tai Yu

**Affiliations:** 1 Department of Neurology and National Center for Neurological Disorders, Huashan Hospital, State Key Laboratory of Medical Neurobiology and MOE Frontiers Center for Brain Science, Shanghai Medical College, Fudan University, National Center for Neurological Disorders, 12Th Wulumuqi Zhong Road, Shanghai, 200040 China; 2 Department of Neurology, Qingdao Municipal Hospital, Qingdao University, Qingdao, China; 3 Department of Rehabilitation Medicine, Huashan Hospital, State Key Laboratory of Medical Neurobiology, Institute for Translational Brain Research, MOE Frontiers Center for Brain Science, MOE Innovative Center for New Drug Development of Immune Inflammatory Diseases, Fudan University, Shanghai, 200032 China; 4 Institute of Pediatrics, Children’s Hospital, Institutes for Translational Brain Research, State Key Laboratory of Medical Neurobiology, MOE Frontiers Center for Brain Science, Fudan University, Shanghai, 200032 China

**Keywords:** Microglia, Alzheimer’s disease, Neuroinflammation, MS4A6A, Ms4a6d

## Abstract

**Background:**

Alzheimer’s disease (AD) is the most common type of dementia. Genetic polymorphisms are associated with altered risks of AD onset, pointing to biological processes and potential targets for interventions. Consistent with the important roles of microglia in AD development, genetic mutations of several genes expressed on microglia have been identified as risks for AD. Emerging evidences indicate that the expression of a microglia-specific gene *MS4A6A* is thought to be associated with AD, since AD patients show upregulation of *MS4A6A*, and its levels correlate with the severity of clinical neuropathology. However, the mechanism linking MS4A6A and AD has not been experimentally studied.

**Methods:**

We performed a meta genome-wide association analysis with 734,121 subjects to examine the associations between polymorphisms of *MS4A6A* with AD risks. In addition, we analyzed the correlation between MS4A6A and AD-related cerebrospinal fluid biomarkers from our own cohort. Furthermore, we for the first time generated a Ms4a6d deficient APP/PS1 model, and systematically examined pathological changes using high-resolution microscopy, biochemistry, and behavioral analysis.

**Results:**

We identified several new mutations of *MS4A6A* with altered AD risks, and discovered specific correlation for some of them with the amount of β-amyloid in cerebrospinal fluid. Protective variant of *MS4A6A* is associated with elevated expression of the gene. Deficient Ms4a6d led to reduced amyloid clearance in the brain. Immunostaining from postmortem AD patients brain revealed selective expression of MS4A6A in microglia. In APP/PS1 mice lacking Ms4a6d, microglia showed markedly diminished envelopment and phagocytosis of amyloid, leading to increased plaque burden, less compact structure, and more severe synaptic damage. Importantly, Ms4a6d deficiency markedly exacerbated inflammatory responses in both microglia and astrocytes by disinhibiting NF-κB signaling. Overexpressing MS4A6A in human microglia cell line promoted gene expression related to plaque-associated responses and diminished inflammation signatures.

**Conclusions:**

Our findings reveal that Ms4a6d deficiency suppresses neuroprotection and worsens neuroinflammation. Sufficient Ms4a6d maybe beneficial for boosting amyloid-related responses and suppressing inflammation in microglia, making it superior than previously reported candidates for microglia modulation. Thus, the elevated MS4A6A levels in AD are likely compensatory and boosting MS4A6A could be an effective treatment.

**Supplementary Information:**

The online version contains supplementary material available at 10.1186/s13024-025-00887-0.

## Background

Alzheimer's disease (AD) is the most common cause of dementia, while therapeutic benefits from current treatment strategies are still quite limited [1, 2]. Numerous single nucleotide polymorphisms (SNPs) have been identified to be associated with changes in risks of developing AD [3, 4], which provide molecular candidates for understanding the mechanism of AD pathogenesis. Among genetic variations associated with AD, rs610932 locates on the *MS4A6A (Membrane spanning 4-domains A 6 A)* gene and previous studies identified this variant is linked with reduced AD risks in Caucasians [5–7] and Asians [8]. In addition, rs610932 is also correlated with decreased rate of brain atrophy in middle temporal cortex and entorhinal cortex [9, 10]. Furthermore, another SNP rs7232 on the *MS4A6A* gene shows a congruent protective effect [11], corroborating the involvement of this gene in the process of AD pathogenesis.

However, the mechanism linking *MS4A6A* to AD is not known. *MS4A6A* belongs to the *MS4A (Membrane spanning 4 A)* gene family, which is a group of four-transmembrane-domain proteins with variable cytoplasmic regions. Among them, MS4A6A and MS4A4A are highly expressed in brain tissue. Previous work demonstrated that *MS4A6A* is selectively expressed in microglia [12–14], and stimulation by amyloid pathologies leads to elevated expressions [15–17]. Consistently, elevated *MS4A6A* expression is correlated with higher Braak stages in human brains [18], and MS4A6A levels in periphery blood are significantly higher in AD patients compared to controls [19]. Based on these data, it has been proposed that elevated levels of MS4A6A might be detrimental [19], yet this has not been experimentally tested.

Due to its expression specificity, MS4A6A likely affects microglia function. Microglia play complex and important roles in the pathogenesis of AD. Several genetic mutations of microglia-specific genes have been identified as risks for AD, including TREM2 (Triggering receptor expressed on myeloid cells 2) [20, 21], CD33 [22] and MS4A [5, 8]. One prominent pathological feature in AD brain is the presence of reactive microglia [23]. These microglia appear to be inflammatory [24] with cytotoxicity [25], which contribute to the degenerative process in AD. On the other hand, as the resident immune cells in the brain, microglia are crucial for clearing β-amyloid (Aβ) via phagocytosis [26, 27]. And recently it has been reported that amyloid plaque-associated microglia could be neuroprotective by encasing neurotoxic Aβ with a physical barrier [28–32], which prevents the accumulation of protofibril amyloid materials around plaques and reduces the development of axonal spheroids pathologies [33]. Previous analysis revealed positive correlation between the expression levels of *MS4A6A* and *TREM2* in human brains [11, 12]. And its mouse homolog Ms4a6d has been shown to act as an inflammation suppressor in periphery macrophages [34]. These studies suggest potential regulation of microglia function by MS4A6A yet direct investigation of its function in the context of AD has not been done.

In this study, we established a mouse model with *Ms4a6d* knockout in the APP/PS1 background. We provided evidence that *Ms4a6d* deficiency exhibit diminished microglia wrapping around plaques and reduced phagocytosis, at the same time showed extensive activation of inflammatory pathways. For the first time, our data provide a plausible mechanism underlying the *MS4A6A* mutations’ link to AD onset. And the striking divergent effect of *Ms4a6d* implies that activation of this target could be beneficial from both aspects of the microglia functions which is ideal for AD interventions.

## Methods

### Participants

#### CABLE cohort

Chinese Alzheimer’s Biomarker and LifestylE (CABLE) was a large population cohort recruited from Qingdao Municipal hospital, including risk factors and biomarkers of AD [35]. The participants are between 50 and 90 years old. Based on their educational level and the results of the Montreal Cognitive Assessment (MoCA), participants were classified as normal cognition and mild cognitive impairment (MCI). Illiterate individuals with MoCA values lower than 13 were classified as MCI. For individuals received 1 to 6 years of education, the cutoff value is 19. And for individuals received education over 7 years, the cutoff value is 24. Participants diagnosed with any of the following diseases will be excluded: (1) major neurological disorders such as multiple sclerosis, epilepsy, head trauma, central nervous system infections; (2) serious psychological disorders; (3) severe systemic diseases like cancer that could influence AD biomarkers levels; and (4) familial hereditary genetic disease. Biochemical tests, neuropsychological tests, and the collection of blood and cerebrospinal fluid (CSF) samples were all performed routinely. An electronic medical record system and an extensive questionnaire were used to gather data on medical history, demographics, and AD risk factor profile. The CABLE study was carried out following the Declaration of Helsinki and the Qingdao Municipal Hospital's Institutional Ethics Committees approved the study's protocol. Participants or their guardians provided the written informed consent.

In this study, 990 elderly adults were included from the CABLE database for analysis. The demographic characteristics of participants were detailed in Supplementary Table S1.

#### The UK biobank

The UK Biobank is a prospective study that includes 500,000 participants and detailed documentations of baseline characteristics, disease risk factors, past medical history, and long-term follow-up from connected medical data. At the baseline assessment, every participant electronically signed a statement of informed consent. The baseline period of the study was from 2006 to 2010, and participants between the ages of 37 and 73 were followed until their first record of AD diagnosis, death, loss to follow-up, or the last date on which data were still available (November, 2021). The UK biobank research was carried out under the approvement of the National Information Governance Board for Health and Social Care and the North West Multicenter Research Ethics Committee. The current analyses were conducted under UK Biobank application number 19542.

#### FinnGen

The FinnGen study (https://www.finngen.fi/en) integrates genome and digital healthcare data. All participants provided informed written consent. Detailed quality control criteria can be found in previous study [36].

### CSF biomarker assessments

All CSF samples in CABLE cohort were collected by lumbar puncture. The CSF was then processed or stored after instant centrifugation at 2000 × g for 10 min. The thaw/freezing cycle for each sample was limited twice. CSF sTREM2 concentrations were determined with the ab224881 Human TREM2 SimpleStep ELISA (Enzyme-linked immunosorbent assay) kit from Abcam. CSF Aβ concentrations were determined with INNOTEST® β-Amyloid (1–42) 81583 and INNOTEST® β-Amyloid (1–40) 80462 from FUJIREBIO. P-tau was measured with INNOTEST® PHOSPHO-TAU (181p) 81581 from FUJIREBIO. Total tau was measured with INNOTEST® hTAU-Ag 81579 from FUJIREBIO. Progranulin was detected using the RMEE103R Progranulin Human ELISA kit (Biovendor, Heidelberg, Germany). α-Synuclein was measured via LEGEND MAX™ Human α-Synuclein ELISA Kit with pre-coated plate (No:844101, BioLegend). All biomarker indexes were measured twice by skilled operators who were blinded to clinical data. For these detected proteins, intra-batch variance for technical replicates was < 5%. The inter-batch coefficients of variations were < 20%.

### Associations between CSF biomarkers and the SNP

Samples were genotyped using the Infinium Asian Screening Array Kit on the Illumina BEADLAB platform. Standard quality control was then performed, as described in our previous study [37]. Variants located within the *MS4A6A* gene (chr11:59,939,080–59,952,139 according to GRCh37/hg19) were extracted using the PLINK 2.0 software [38]. An additive linear regression in PLINK 2.0 was applied to investigate the effects of genotypes on CSF biomarkers, adjusted with age, sex, the first two principal components as covariates. The suggestive and genome-wide significant thresholds were *P* < 1 × 10^–5^ and *P* < 5 × 10^–8^, respectively [39].

### Meta-analysis

Three sources of genome-wide association studies (GWAS) data were used for this meta-GWAS analysis, including the UK Biobank database, and Kunkle stage1 [6], as well as data from the FinnGen consortium [36].

The imputed SNP genotype data employed in this study were based on the GRCh37/hg19 build [40]. In UK Biobank, we excluded participants who were not of European descent, those with a missing genotype rate exceeding 0.05, or a mismatch data on sex (Data field 31 and 22,001), those with chromosomal disorders, heterozygosity rate outliers, more than ten putative third-degree relatives, and those who are not utilized in computing the principal components. Additionally, multi-allelic variants and call rate < 0.95, a minor allele frequency < 0.01, Hardy–Weinberg* P*-value < 10^–6^, or imputation quality score < 0.5 were filtered out. For subsequent analysis, we focused on variants specifically located in the *MS4A6A* gene. Sample whose age at disease onset or last follow-up was younger than 65 years old were removed. Following the quality control process, a total of 3,076 AD cases and 254,938 controls with available phenotype and covariates data were retained for further investigation. GWAS was performed to test the correlation between variants in *MS4A6A* and AD risk using logistic regression controlled for age, sex, batch, and the first three principal components in PLINK2.0 [38]. Furthermore, we obtained publicly accessible summary-level data from Kunkle stage 1 (21,982 AD cases and 41,944 controls) and FinnGen consortium (10,520 AD cases and 401,661 controls).

GWAMA software was used to combine summary statistics from the three studies [41].

### MS4A6A Overexpression

Human microglial cells (HMC3) were maintained in MEM supplemented with 10% FBS at 37 °C with 5% CO₂. For transduction, cells were seeded at 5 × 10^5^ cells/well in 6-well plates and infected with *MS4A6A* overexpression lentivirus rLV-CMV-hMS4A6A-3xHA-P2A-EGFP-hPGK-Puro-WPRE at MOI 15 in the presence of 6 μg/mL Polybrene. After 24 h, the medium was replaced with fresh complete medium. Selection with 2 μg/mL puromycin commenced at 72 h post-infection and continued for 10 days until non-transfected controls showed complete mortality.

### RNA Sequencing and analysis

Target cells were harvested. Total RNA was isolated with FreeZol Reagent (Vazyme). Libraries were prepared using the Illumina TruSeq Stranded mRNA Kit and subsequently used for RNA-seq analysis. High-quality reads were aligned to the mouse reference genome using Bowtie2. Expression levels for each of the genes were normalized to fragments per kilobase of exon model per million mapped reads using RNA-seq by Expectation Maximization. DEGs (differentially expressed genes) between samples were identified and performed clustering analysis and functional annotation. Differential expression analysis was conducted in DESeq2 (P < 0.05, |log2FC|> 0.5). Gene ontology (GO) and KEGG pathway enrichment were analyzed using ClusterProfiler v4.2.2. Three biologically independent replicates were included for each experimental group.

### Construction of plasmids and stable point mutation cell lines

We generated the base editing plasmid used for *MS4A6A* related SNP mutation. For rs646924: T > C (A > G) mutation, the adenine deaminase/cytidine deaminase MU148 described previously was inserted into the internal 1,249 site of the nickase Cas9 variant SpG [42]. sgRNA was cloned into the pU6-sgRNA-EF1α-UGI-T2A-PuroR plasmid linearized with BsaI; paired oligonucleotides were synthesized, annealed and inserted to construct the sgRNA expression plasmid. The sgRNA sequence used for the rs646924: A > G mutation was 5’-ggcaagggaatagtgagatg-3’.

Human microglial cells were seeded at 600,000 cells per well on 6-well plates. The following day, 1500 ng of the base editor expression plasmid was co-transfected with 500 ng the sgRNA expression plasmid into human microglial cells using the jetPRIME® transfection reagent (Polyplus-transfection) following the manufacturer’s instructions. 24 h post-transfection, the complete medium was replaced with a fresh complete medium containing 2 μg/ml puromycin every day for 4 days. Next, cells were trypsinized and resuspended in complete medium and then sorted into a 96-well plate containing complete medium. Surviving single-cell colonies were transferred into the 12-well plates and cultured for another 9–10 days. After cell expansion, the genome DNA of each single-cell clone was extracted for targeted amplification to examine the mutation status. Cell clones containing the expected SNP mutations were used for functional studies.

### qPCR

Total RNA was isolated from edited cultured microglia and unedited control using FreeZol Reagent (Vazyme) according to the manufacturer’s protocol. RNA concentration and purity were determined spectrophotometrically (NanoDrop). After DNase I treatment, 1 μg RNA was reverse-transcribed into cDNA (HiScript III RT SuperMix for qPCR, Vazyme) with oligo(dT) primers. qPCR reactions were performed in triplicate on QuantStudio 5 (Applied Biosystems) using ChamQ Universal SYBR qPCR Master Mix (Vazyme) in 20 μL total volume. Relative expression was normalized to *GAPDH* reference genes and calculated using the 2^(-ΔΔCt) method. Primer sequences for target genes (e.g., MS4A6A: F 5′-TGTGGCATGATGGTATTGAGC-3′, R 5′-AGGGTCCTATGAATGGGTAAGC −3′; GAPDH: F 5′-GAAGGGCATCTTGGGCTACAC-3′ R 5′-GTTGTCATTGAGAGCAATGCCA-3′).

### Animals

The Ms4a6d^−/−^ mice were generated by Cyagen Bioscience using the CRISPR/Cas9 technique. Exon 2–7 of *Ms4a6d* were knocked out. APP/PS1 mice were purchased from Aniphe Biolaboratory and crossed with Ms4a6d^−/−^ mice to develop Ms4a6d^−/−^ under APP/PS1 mice background (termed throughout the paper APP/PS1: Ms4a6d −/−, APP/PS1: Ms4a6d +/−, and APP/PS1: Ms4a6d +/+). The genotype of mice was confirmed by Sangon Biotech. Age-matched wild type mice were bought from Vital River Laboratory Animal Technology. In the animal center of Huashan hospital, mice were housed in rooms with temperature and humidity controls (21 ± 1.5℃ and 50 ± 10%) under conventional 12-h light/dark cycle settings. The mice were kept in an environment free of pathogens. All of the experiments comply with the animal study protocol approved by the Fudan University.

### Human postmortem brain tissues

The midbrain tissue used in this study was collected from an 89 years old female sporadic Parkinson's disease patient. This patient was autopsied after death and pathologically diagnosed with Lewy body pathology found in the substantia nigra region. The formalin-fixed, paraffin-embedded specimen was a gift from Body Organ Donation Center of Dalian Medical University. The consent for body and organ donation (code number: 00303) was carefully signed by the donor. Her next of kin have provided patient's basic information and clinical history.

### Stereo-seq

A total of nine C57BL6/J mice (three for each age group) were initially housed at Jiangsu Cavens Biolaboratory Inc., and maintained at two weeks younger than their designated sacrifice ages: postnatal 2 months (8 weeks), 6 months (24 weeks), and 12 months (48 weeks). Subsequently, they were transferred to Daoke Pharmacology Inc. Facility for a minimum of one week to acclimate prior to sacrifice. Mice were group-housed and, prior to trans-cardial perfusion, anesthetized using 4% isoflurane via face mask. The collected whole brains were washed with cold phosphate-buffered saline, dried, and then embedded in optimal cutting temperature (O.C.T.) compound. Brains were cryosectioned at −20°C on a cryostat and immediately snap-frozen at −80 °C. Stereo-seq (SpaTial Enhanced REsolution Omics sequencing) for the spatial trasncriptomic analysis were prepared as described before [43]. In total, three forebrain sections (interaural 3.94 mm, bregma 0.14 mm) and three hindbrain sections (interaural 1.50 mm, bregma −2.30 mm) of 10 μm thickness were collected at each age group, and these sections were promptly adhered to the surface of Stereo-seq chips at −20°C. Tissue sections were fixed in cold methanol for at least 40 min at −20°C and washed three times in 1 × saline sodium citrate before proceeding with Stereo-seq library preparation. All of the procedures were conducted with the approval of the Institutional Animal Care and Use Committee of Fudan University (approval number: JS-256).

### In vivo Aβ Microdialysis

Microdialysis probes with a 2 mm, 100 kDa molecular weight cutoff membrane (CMA 8 microdialysis probes) were stereotaxically implanted into following the coordinates for left hippocampus (bregma: −3.0 mm, 2.5 mm lateral to midline, and 4.0 mm below dura). Perfusion buffer (0.15% bovine serum albumin in artificial cerebrospinal fluid (in mM: 1.3 CaCl_2_, 1.2 MgSO_4_, 3 KCl, 0.4 KH_2_PO_4_, 25 NaHCO_3_, and 122 NaCl, pH7.35) was perfused at a 1-µl/min flow rate with a syringe pump (CMA). Microdialysates were collected every 60 min at 4 °C using a refrigerated fraction collector. Mice was anesthetized with isoflurane inhalation during the process.

Interstitial fluid (ISF) human Aβ_1-x_ concentrations were quantified hourly via ELISA (No.27729, IBL). Baseline Aβ_1-x_ levels were defined as the mean concentration from hours 3–5 after probe insertion. Then ISF was obtained for 8 h after intraperitoneally injecting each mouse with 6.7 mg/kg of the γ-secretase inhibitor Compound E (Med Chem Express). Aβ_1-x_ values at each time point were expressed as percentage of baseline mean (% baseline).

### Preparation of brain sample

All animals received transcardial perfusion with ice-cold phosphate-buffered saline (PBS) after being anesthetized with isoflurane inhalation. The 4% paraformaldehyde fix solution was utilized to fix the right hemispheres for a whole night at 4 °C. Fixed mice brain tissues were sectioned at 45 μm-thick on a vibratomes (Leica, Leica VT1000S). The left hemisphere's cortex and hippocampus were carefully dissected out, then quickly frozen in liquid nitrogen for further biochemical testing.

### Immunohistochemistry

Mouse sections were rinsed with PBS three times, then blocked with 5% bovine serum albumin in PBS and permeabilized with 0.5% Triton-X 100 in blocking solution. To ensure adequate antigen retrieval, the human specimen was heated at 95 °C in the 10 mM citrate buffer (pH 6.0) for 40 min and washed for 5 min three times after cooling down at room temperature.

Tissues were first incubated with corresponding primary antibodies overnight: Iba1 (1:100, ab5076, Abcam) or (1:500, 019–19741, Wako), Lamp1 (1:200, 1D4B, DSHB), Phospho-Jak2 (Tyr1007/1008) (1:100, 3771S, Cell Signaling Technology), 4G8 (1:1000, 800,701, BioLegend), Phospho-NF-κB p65 (Ser536) (1:1000, 3033S, Cell Signaling Technology), GFAP (1:500, ab4674, Abcam), CD68 (1:200, MCA1957, Bio-rad), MS4A6A-N-terminal (1:50, ab189983, Abcam). Secondary antibodies were chosen according to primary antibodies host species: anti-goat IgG Alexa-Fluor555 (1:500, Invitrogen), anti-mouse IgG Alexa Fluor 488 (1:500, Invitrogen), anti-rabbit IgG Alexa Fluor 647 (1:500, Invitrogen), anti-rat IgG Alexa Fluor 555 (1:500, Invitrogen), anti-chicken IgG Alexa-Fluor 488 (1:500, Invitrogen) for 1 h at room temperature at dark. Thioflavin S (Sigma-Aldrich, 2 mg/10 mL) was used to stain plaques for 25 min. Then sections were washed for 5 min three times before mounting on slides with Fluoromount-G® mounting medium or DAPI Fluoromount-G® mounting medium (SouthernBiotech).

### Fresh frozen tissue preparation

Adult C57BL/6 J mice were anesthetized with isoflurane. Brains were harvested within 5 min post-sacrifice and maintained on ice. For embedding, tissues were briefly blotted on sterile paper towels, oriented in cryomolds, and covered with O.C.T. compound. Molds were rapidly frozen on a liquid nitrogen platform for 10 min until O.C.T. solidified. Continuous coronal sections were made at −20 °C using a constant cooling microtome (Leica) with a thickness of 20 μm. The sections were adhered to slides treated, dried at room temperature for 30 min, and then stored at −80 °C for long-term preservation.

### RNA-scope

RNA in situ hybridization was processed according to the manufacturer's protocol for the RNA-scope Multiplex Fluorescent Reagent Kit v2 (Advanced Cell Diagnostics). The sections were dehydrated in a gradient of 70%, 50%, and 100% ethanol, then incubated with hydrogen peroxide at room temperature for 10 min, and washed twice. Target-specific probes were hybridized sequentially: mouse Aif1-C2 (433,091, Advanced Cell Diagnostics) and Ms4a6d (314,591, Advanced Cell Diagnostics) probes were applied and incubated at 40 °C for 2 h in a HybEZ™ hybridization oven and wash twice. Post-hybridization signal amplification was performed through 2-step amplifier reactions (AMP1-2) as per kit instructions. Tyramide Signal Amplification (TSA) was subsequently conducted using the TSA Plus Cyanine Evaluation Kit, with fluorophore-conjugated tyramide (1:750, Advanced Cell Diagnostics) incubated at 40 °C for 30 min protected from light. Then sections were mounted on slides with DAPI Fluoromount-G® mounting medium (SouthernBiotech).

### Confocal microscopy

All confocal images were collected through an Olympus system. More than 20 plaques from each mouse were taken for all genotypes. For each staining set, the setting of laser and detector were constant. Using a 60 × oil lens, graphs were captured at 1024 × 1024 pixels with 4 × zoom and a 1 μm z-step. Images were processed with ImageJ or Matlab (Mathworks). Maximum intensity projection of the z-stack for each graph was used for morphological analysis. During analysis, the mice and group data were kept blind.

Plaque shapes were identified using a threshold fluorescence intensity on the Thioflavin S channel that was two standard deviations above the average fluorescence intensity of the image. Activated microglia processes tightly encased amyloid plaques, with Iba1 (Ionized calcium binding adapter molecule 1) expression higher than baseline microglial processes, were characterized as the microglia barrier surrounding compact plaques. The Iba1 fluorescence intensity in the cell body was used as the cutoff value to threshold the Iba1 channel. The percentage of the plaque perimeter covered by the microglia barrier was ascertained by calculating the angle between contact points from the plaque center.

For analysis of the staining of Iba1, CD68, 4G8, phosphorylated JAK2 (Janus kinases 2) and phosphorylated p65, a z-projection of maximal fluorescence intensities across 10 optical slices through the center of the plaque core was made. The intended fluorescent channel was thresholded using “Default” preset in FIJI and fluorescent colocalization analysis were performed using “AND” function for colocalization.

For measuring amyloid plaque distribution, the Thioflavin S fluorescent channel was thresholded using “Triangle” present in FIJI and those plaques were measured using “Analyze Particle” function. The size and intensity of each particle was recorded and analyzed together. The total plaque area was calculated according to the percentage in each region (cortex, hippocampus, thalamus, basolateral amygdala).

Axonal spheroids areas were calculated by thresholding the Lamp1 (Lysosome associated membrane protein 1) channel image with 150% average fluorescence intensity of the background after subtracting the area of plaque in a 10-slice z-projection through the center of plaque core.

Multichannel fluorescence images were acquired on an Olympus confocal system equipped with a 60 × oil-immersion objective. Z-stack images (1 μm step) were processed with maximum intensity projection in ImageJ. Assays were typically performed in parallel with positive and negative controls, to ensure interpretable results. To quantify the RNA expression signal, the fluorescent dots of *Aif1* and *Ms4a6d* within the DAPI-stained cell nuclei in the field of view were recorded. The presence of *Aif1* expression indicated the microglia. The proportion of microglia among all the cells expressing *Ms4a6d* was calculated.

### Immunoblotting

Brain tissues were lysate by buffers with Protease Inhibitor Cocktail (Beyotime) and boiled with 5 × SDS loading buffer (Beyotime). The protein level of each sample was measured by BCA kit (Beyotime) and each sample was then diluted into the same level before loading. 25ug per lane was the standard protein amount for western blotting in this study. Antibodies and dilutions used in this study include: NLRP3 (1:1000, AG-20B-0014, Adipogen), β-tubulin (1:2000, 10,068–1-AP, Proteintech), PSD95 (1:2000, ab238135, Abcam), Synaptophysin (1:2000, 36406S, Cell Signaling Technology), β-actin (1:2000, 20,536–1-AP, Proteintech). Quantitative analyses were performed on ImageJ software.

### ELISA assay

The concentrations of mouse IL-1β (Interleukin-1β) in cortical lysates were tested with ELISA kits (FineTest) under standard procedures. Total protein level of each sample was measured by BCA kits (Beyotime). Samples were measured in duplicates.

### Novel object recognition task

The novel object recognition task is carried out in an open field arena using two different types of objects, cylinder and cuboid. During habituation, the animals were free to explore an empty arena for 5 min. Next day, the animals were brought back to the same arena where mice could investigate two identical objects placed at equal distances from the edge for 10 min. Mouse who had a preference of one side was excluded from further testing. 24 h later, mice were then tested for long-term identification recall by allowing them to wander the same field with the familiar object and a novel object in 10 min. The objects and arena were thoroughly cleaned between each mouse experiments. Mice whose total exploration time was less than 20 s were excluded. The time spent exploring each object, as well as the investigation ratio (novel object exploration time/(novel object exploration time + familial object exploration time)) were recorded. Trajectory of each mouse was tracked with DeepLabCut^TM^ and analyzed with MATLAB (MathWorks).

### Statistics

The data were shown as mean ± S.E.M. (Standard Error of Mean). For comparisons between two groups, two-tail unpaired Student’s t test was used. For multiple group comparisons, one-way ANOVA tests was employed with post hoc Tukey tests. All statistical analysis were completed on Prism (GraphPad). *P* values less than 0.05 were regarded significant in this study. *P < 0.05, **P < 0.01, ***P < 0.001, ****P < 0.0001, ns = not significant.

## Results

### *MS4A6A* SNPs modulate Aβ pathologies and risks for human AD

**Fig. 1:**
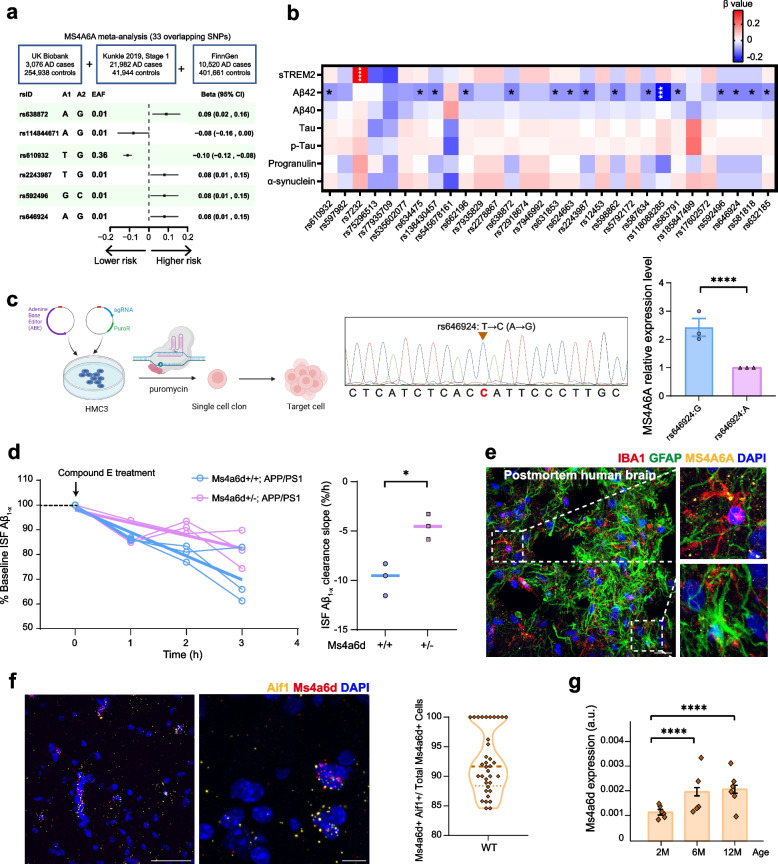
Human *MS4A6A* SNPs are correlated with AD risks and amyloid biomarkers in the CSF. **a** Meta-analysis of the genome-wide associations between *MS4A6A* SNPs and risks of AD. Three cohorts were included: UK Biobank, Kunkle Stage 1 and FinnGen consortium. Error bars indicate the upper and lower boundaries of confidence interval. A1 indicates the effect alleles with the frequencies listed under effect allele frequency (EAF). A2 shows the control alleles. Generalized linear regression models were used for statistical comparisons. **b** Heatmap of association between *MS4A6A* SNPs and neuropathological biomarkers in CSF. Generalized linear regression models were used for statistical comparisons. Significance is shown on the image: blank, *P* > 0.05, *, *P* < 0.05, ***, *P* < 0.001, ****, *P* < 0.0001. **c** Left: Workflow diagram of rs646924: T > C (A > G) editing via single-base editing in HMC3 cells followed by monoclonal cell line isolation. Middle: Validation of rs646924: T > C editing by Sanger sequencing in monoclonal derivatives. Right: qPCR analysis of MS4A6A expression at the rs646924: G locus versus controls rs646924: A (normalized as 1). Two-tail unpaired student’s t-test was used. ****, P < 0.0001.* N* = 3 repeated experiments. **d** ISF Aβ_1-x_ obtained by microdialysis from 1-month-old APP/PS1:Ms4a6d +/+ mice (blue) and APP/PS1:Ms4a6d +/− mice (magenta). Baseline Aβ_1-x_ levels were defined as the mean concentration from hours 3–5 after probe insertion. Aβ_1-x_ values after γ-secretase inhibitor Compound E treatment at each time point were expressed as percentage of baseline mean (% baseline). Raw data were fitted with linear trend lines. Two-tail unpaired student’s t-test was used. *, *P* < 0.05. *N* = 3 mice for each group. **e** Left panels show representative confocal images of MS4A6A (yellow) co-stained for IBA1 (red, for microglia) and GFAP (green, for astrocytes) in human brain tissues. Right panels show zoomed microglia and astrocyte images from the boxes in the left. Scale bar: 20 µm. **f** Representative RNA-scope images and quantification of RNA transcripts of *MS4A6A* (red) and microglia marker *Aif1* (yellow). Nuclei were stained with DAPI. Right panel shows the quantification of *Ms4a6d*-positive microglia in total *Ms4a6d* -positive cells. Left panel scale bar: 50 µm. Middle panel scale bar: 10 µm. *N* = 3 mice. **g** Quantifications of Ms4a6d expression level in wild type mice at 2-month, 6-month and 12-month based on stereo-seq data. One-way ANOVA tests with post hoc Tukey tests were used. ****, *P* < 0.0001. a.u., arbitrary unit. *N* = 6 mice

Previous GWAS studies identified that the T (minor) allele of the rs610932 locus of the *MS4A6A* gene is associated with a reduced risk for AD [7]. Subsequent studies verified the involvement of this gene in the development of AD in additional Han Chinese and Caucasian cohorts [5, 8, 44]. To further examine the association of other *MS4A6A* SNPs with AD, we analyzed data using UK Biobank, Kunkle stage 1, and FinnGen cohorts. We identified total 33 overlapping SNPs between these cohorts and 19 of them showed congruent effect among these three studies (I^2^ = 0%). We were able to replicate previously reported associations between minor alleles at rs610932 (T allele), rs7232 (A allele), rs583791 (C allele) and lower AD risk. Notably, most of these SNPs belong to a group with high linkage disequilibrium (rs7946992, rs7935829, rs2278867, rs12453, rs17602572, rs72918674, rs7232, rs662196, rs634475, rs631853, rs583791, rs632185, rs610932, rs624663) (Supplementary Fig. S1), of which the minor allele all showed association with decreased risk for AD. We further found that four new SNPs (rs638872, rs2243987, rs592496, rs646924) were AD risk loci and one SNP (rs114844671) was protective (Fig. 1a). Together, these results revealed several new SNPs of *MS4A6A* related to AD, suggesting potentially important roles of *MS4A6A* gene in AD pathogenesis.

In order to further investigate the mechanisms underlying these associations, we collected CSF samples from individuals with and without *MS4A6A* mutations in the CABLE cohort (Supplementary Table S1) and measured the levels of AD-related biomarkers. We identified 31 SNPs of the *MS4A6A* gene in our cohort, 28 of which have not been previously studied in the context of AD. We found that the majority of *MS4A6A* SNPs showed significant associations with reduced CSF Aβ42 levels, after controlling for the effects of age and gender (Fig. 1b). While decreased levels of Aβ42 in CSF are often related to increased amyloid deposits in the brain [45, 46], the consistent effects on Aβ42 could also be indications of enhanced amyloid clearance. Importantly, SNPs in the *MS4A6A* gene seems to selectively affect AD-related pathologies, since we did not observe significant correlation between *MS4A6A* SNPs and progranulin or α-synuclein levels, and only one SNP (rs7232) correlated with increased soluble TREM2 levels as previously reported [11, 12]. These data strongly suggest that *MS4A6A* gene likely modulates AD pathogenesis via affecting amyloid pathologies, which we directly examined in vivo.

It is worth noting that all the SNPs reported above locate outside the exon regions of the gene, thus they likely influence disease risk by modulating the expression levels of *MS4A6A*. To directly test this, we engineered isogenic point mutation rs646924: T > C (A > G) in human microglia cell line (HMC3) using CRISPR-Cas9 (Fig. 1c). We found that rs646924 A allele (risk allele) significantly reduced expression of *MS4A6A* compared to the G allele (Fig. 1c). These results suggest that decreased *MS4A6A* transcription could lead to increased AD risk, likely via affecting amyloid-related processes.

To examine the effect of *MS4A6A* deficiency, we aimed to establish a *MS4A6A* knockout mouse model. However, the human *MS4A6A* gene is not present in mice. Previous studies have identified *Ms4a6d* in mice as a homologue gene for human *MS4A6A* [34, 47]. We examined the protein sequence and predicted structures of human MS4A6A and the MS4A gene family in mice, and found that Ms4a6d showed the highest similarity to human MS4A6A (Supplementary Fig. S2 and Supplementary Table S2). Therefore, we generated a *Ms4a6d* knockout mouse model (Supplementary Fig. S3). To directly measure the impact on amyloid-related processes, we cross-bred the Ms4a6d knock-out mice with AD-like transgenic line APP/PS1, and performed intracerebral microdialysis to assess Aβ clearance efficiency in 1-month-old mice before the formation of amyloid deposits. After administering γ-secretase inhibitor to block Aβ production, we observed a decline of Aβ_1-x_ levels in interstitial fluid (ISF), indicating the rate of Aβ clearance. We found that the rate of Aβ clearance was slower in Ms4a6d-deficient mice compared to control APP/PS1 mice (Fig. 1d). These results demonstrated that Ms4a6d deficiency impairs Aβ clearance in the brain.

### Microglia lacking Ms4a6d show diminished response to amyloid plaques

We next examined the cellular mechanism underlying the modulation of Aβ depositions by *MS4A6A* gene. Previous studies indicated that the MS4A6A is selectively expressed in microglia in the brain [47]. We further examined the expression pattern of MS4A6A with human postmortem brain tissues through immunofluorescence staining. Consistent with previous reports, confocal images showed that human MS4A6A was specifically enriched within microglia but not in astrocyte (Fig. 1e). Thus, we hypothesized that MS4A6A may be an important regulator for microglia functions. We also tested the expression pattern of *Ms4a6d* with in situ hybridization on mice brain sections. The results showed that above 92% on average, and many times 100%, of the *Ms4a6d* was expressed in microglia. These data indicated that *Ms4a6d* expression is microglia specific (Fig. 1f), consistent with the results in a latest large-scale proteomic study across murine models [48]. In addition, as previous studies identified elevated levels of MS4A6A is associated with risks of AD onset in humans [19] and elevated expression of *Ms4a6d* in APP/PS1 mouse [15], we found that there is an increased expression level of Ms4a6d as mice age (Fig. 1g), which could represent a compensatory mechanism to promote amyloid clearance.

**Fig. 2:**
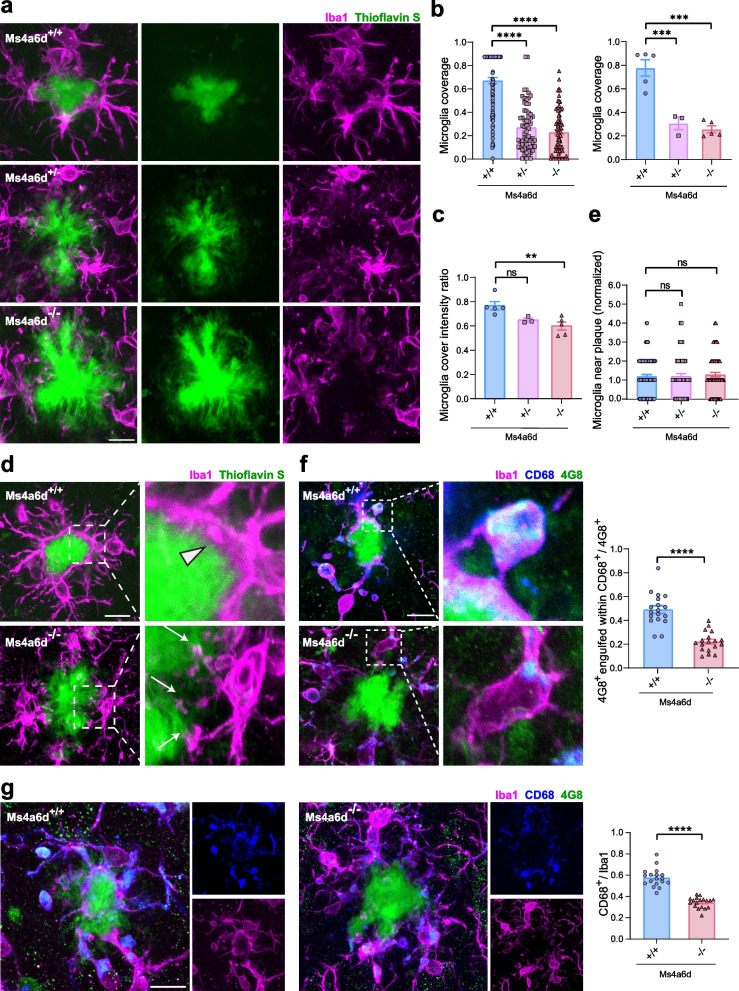
Ms4a6d deletion diminishes microglial responses to Aβ pathology in APP/PS1 mice. **a** Representative confocal images of Iba1-positive microglia (magenta) enveloping the Thioflavin S-positive amyloid plaques (green) in APP/PS1 mice with different *Ms4a6d* genotypes. **b** Quantification of microglia coverage, normalized by 360°. Statistical comparisons were calculated with analysis by individual plaques (left) or by mice (right). Dots in the right panel indicate data from individual animal. **c** Quantification of the Iba1 immuno-staining fluorescent intensities in the near-plaque processes normalized by intensities in the whole cell. **d** Representative zoomed images of microglia processes near amyloid plaques. Microglia from APP/PS1:Ms4a6d +/+ mice closely wrap around plaques (arrow head), whereas microglia from Ms4a6d deficiency mice show dysmorphic processes with looping structures (white arrows). Right panels show further zoomed images from the boxes on the left. **e** Quantification of the number of microglia near plaques, normalized by numbers in APP/PS1:Ms4a6d +/+ mice. **f** Representative confocal images and quantification of Aβ (immunostained with 4G8, green) engulfment within microglial (immunostained with Iba1, magenta) phagosomes (immunostained with CD68, blue). Right panels show further zoomed images from the boxes on the left. **g** Representative confocal images and quantification of CD68-positive vesicular structures in microglia around plaques. Right panels show separate fluorescence channel from the left image. In all panels, data are presented as mean ± S.E.M. Scale bars show 10 µm. *N* = 5 mice for APP/PS1:Ms4a6d +/+, *n* = 3 mice for APP/PS1:Ms4a6d +/−, n = 5 mice for APP/PS1:Ms4a6d -/-. One-way ANOVA tests with post hoc Tukey tests for panels **b**, **c** and **e**. Two-tail unpaired Student’s t-test for panels **f** and **g**. ns, not significant, **, *P* < 0.01, ***, *P* < 0.001, ****, *P* < 0.0001

We next investigated the effect of Ms4a6d deficiency on amyloid-related pathologies. We found that Ms4a6d deletion did not affect the overall distribution and density of microglia (Supplementary Fig. S4a). Thus, we then focused on microglia-plaque interactions. Our previous work demonstrated a neuroprotective function of microglia by wrapping around amyloid plaques [28, 49]. We then examined the distribution and morphology of plaque-associated microglia with or without *Ms4a6d* gene. We found that the deficiency of Ms4a6d led to a marked reduction of microglial envelopment of amyloid deposits (Fig. 2a, b and c). Besides, normal microglia form thickened processes that closely follow the boundaries of amyloid plaques. And in Ms4a6d-deficient microglia, processes failed to enlarge or expand, forming a small bulb at the end of each process (Fig. 2d). While this phenotype is similar to what we reported with Trem2-deficient microglia [28], an interesting difference is that Ms4a6d knockout still exhibits plaque-associated microgliosis at a similar level to Ms4a6d wildtype (Fig. 2e), suggesting that the effect of Ms4a6d deficiency was not related to amyloid sensing, but more likely to affect the downstream signaling or reaction triggered by amyloid.

Another important protective microglia function is the clearance of amyloid via phagocytosis [27]. Thus, we assessed phagocytosis function in plaque-associated microglia by immunostaining the phagosome marker CD68 [50] and the amyloid fragments. We found that microglia lacking Ms4a6d showed reduced intracellular amyloid contents (Fig. 2f), and correspondingly the levels of phagosomes (Fig. 2g). These findings indicate that in the absence of Ms4a6d, both aspects of microglia protective functions are diminished, pointing to a synergized effect to exacerbate amyloid pathology.

### Deletion of Ms4a6d leads to more severe amyloid pathology

**Fig. 3:**
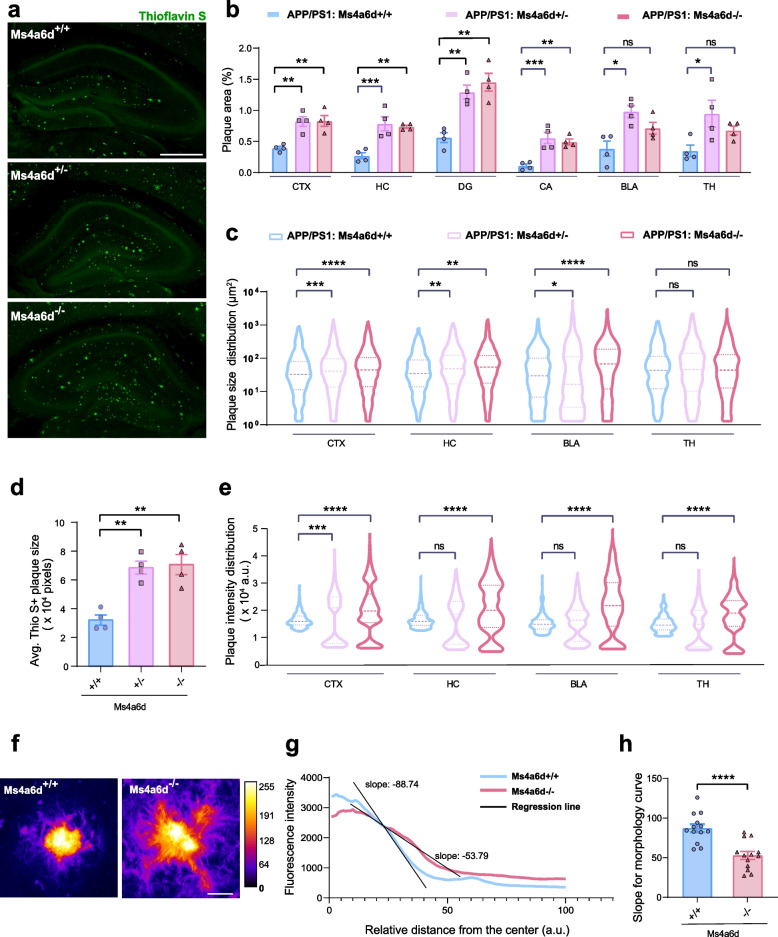
Ms4a6d-deficiency in APP/PS1 mice increases Aβ burden and markedly reduces plaque compaction. **a** Representative images of Thioflavin S staining of plaques in hippocampus area in APP/PS1 mice with different genotypes of Ms4a6d. Scale bar: 500 µm. **b** Quantification of percent area covered by Thioflavin S-positive amyloid plaques in several brain regions of APP/PS1 mice with different *Ms4a6d* genotypes. **c** Violin plots of the size distribution of all individual plaques in several brain regions from APP/PS1 mice with different *Ms4a6d* genotypes. **d** Quantification of amyloid plaques sizes in cortex. **e** Violin plots of individual plaques fluorescence intensity distribution in several brain regions of APP/PS1 mice with different *Ms4a6d* genotypes. **f** Representative heat-map images based on pixel intensity of Thioflavin S staining show distinct morphology of amyloid plaques in the presence or absence of Ms4a6d. Scale bar: 10 µm. **g** Example line graphs of the average Thioflavin S fluorescence intensity changes from plaque centers to the edges. Black lines indicate the linear regression lines for the declining segment from the curves. **h** Quantification of the fitted slopes of the declining segment of fluorescence from plaques of APP/PS1 mice with different *Ms4a6d* genotypes. In all panels, data are presented as mean ± S.E.M. *N* = 4 mice for each group. One-way ANOVA tests with post hoc Tukey tests for panels **b**, **c**, **d**, and **e**. Two-tail unpaired Student’s t-test for panel **h**. ns, not significant, *, *P* < 0.05, **, *P* < 0.01, ***, *P* < 0.001, ****, *P* < 0.0001. CTX, cortex; HC, hippocampus; DG, dentate gyrus; CA, cornu ammonis; BLA, basolateral amygdala; TH, thalamus; a.u., arbitrary unit

Based on the results described above, we examined the impact of Ms4a6d deficiency on amyloid deposition in APP/PS1 mice. We collected brain tissues from 17-month-old mice, and used Thioflavin S staining to label amyloid plaques. Quantification revealed a robust and consistent increase in amyloid depositions in multiple brain regions (Fig. 3a and b). At the same time, the average size of amyloid deposits also enlarged in Ms4a6d-deficient group (Fig. 3c and d). This is consistent with the fact that less amyloid materials were being cleared. In agreement with this view, Ms4a6d-deficient group showed an increase of average fluorescent intensity of Thioflavin S, which is a marker for fibrillar amyloid materials (Fig. 3e) [51]. Interestingly, when we measured the distributions of the fluorescent intensities of plaques, we found that normal mice showed a unimodal distribution of intensities, and Ms4a6d-deficient mice exhibited distributions with multiple peaks, including ones with relatively low intensities (Fig. 3e), suggesting the emergence of diffused amyloid depositions.

Our previous studies indicated that microglia wrapping around plaques leads to compaction of amyloid materials. Following the lead that Ms4a6d deficiency caused reduced microglia wrapping and increased diffuse amyloid plaques, we then assessed the effect of Ms4a6d deficiency on the compactness of amyloid deposits by analyzing the Thioflavin S fluorescence intensity decay from the center of the deposits to the periphery regions (Fig. 3f). Compact plaque structures exhibit well-defined, distinct boundaries between the edge of the labeled amyloid deposits and the surrounding brain tissue, with fluorescence values changing sharply when crossing this boundary. In contrast, loose filamentous structures would exhibit a more gradual decline in fluorescence values along the radius as the labeled fibers extending outwards. In order to quantify this difference, we measured the fluorescence intensity decay from all directions for individual plaques, and calculated the slopes for the averaged trace (Fig. 3g). We found that amyloid deposits in Ms4a6d-deficient mice exhibited a more diffused morphology (Fig. 3h). Overall, consistent with the expected phenotypes from reduced microglial envelopment and phagocytosis of amyloid, Ms4a6d deficiency led to exacerbated amyloid deposits in mice.

### Ms4a6d deficiency exacerbates neuroinflammation and neurotoxicity in APP/PS1 mice

**Fig. 4:**
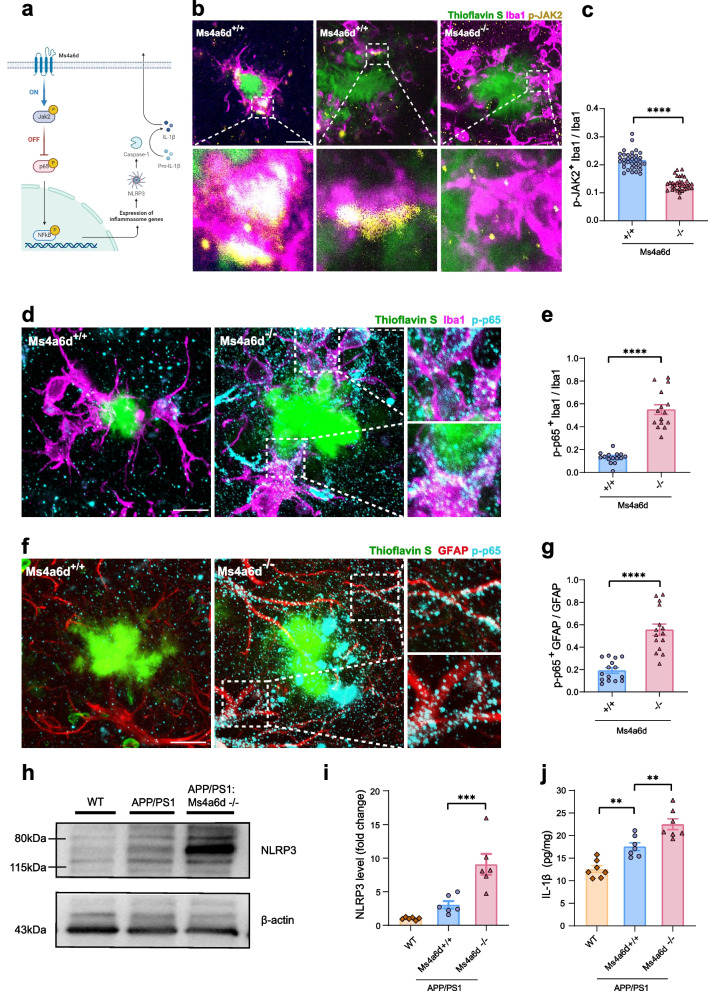
Ms4a6d deficiency dampens JAK2 signaling and promotes neuroinflammation. **a** Diagram of membrane Ms4a6d downstream signaling: Ms4a6d activates JAK2 phosphorylation, leading to the inhibition of p65 phosphorylation, production of NLRP3 and IL-1β. **b-c** Representative images and quantification of immunostaining of phosphorylated JAK2 (yellow) in microglia (magenta) around amyloid plaques (green). Prominent staining can be observed in microglia cell bodies (left) and processes (middle), which is absent in microglia lacking Ms4a6d (right). Lower panels show zoomed images from the boxes above. **d-e** Representative images and quantification of p65 phosphorylation (cyan) in processes adjacent to Aβ deposits (green) and somata of microglia (magenta) in the presence or absence of Ms4a6d. Right panels show zoomed images from the boxes. **f-g** Representative images and quantification of p65 phosphorylation accumulated in astrocytes (immunostained with GFAP, red) in the presence or absence of Ms4a6d. Right panels show zoomed images from the boxes. **h-i** Western blot images of NLRP3 in cortex and associated quantification. The experiments were replicated for three times. Uncropped gels or western blots can be found at Supplementary Fig. S7. **j** IL-1β levels in the cortex from wildtype, APP/PS1:Ms4a6d +/+ or APP/PS1:Ms4a6d -/- mice brains as measured by ELISA. In all panels, data are presented as mean ± S.E.M. Bars: 10 µm. For panels **c, e** and **g**, *N* = 5 mice for each group. *N* = 7 mice for each group in **j**. Two-tail unpaired Student’s t-test for panels **c, e** and **g**. One-way ANOVA tests with post hoc Tukey tests for panels **i** and **j**. *, *P* < 0.05, **,* P* < 0.01, ***, *P* < 0.001, ****,* P* < 0.0001

We next examined the effect of Ms4a6d deficiency in microglia-associated inflammatory responses. Previous studies in periphery macrophages discovered that Ms4a6d is a negative regulator for inflammation [34]. Ms4a6d activates JAK2, which suppresses the downstream NF-κB (nuclear factor κB) activation and subsequent inflammatory responses. We hypothesized that similar signaling pathway exists in the microglia in the brain (Fig. 4a) and examined the presence of phosphorylated JAK2 in control and Ms4a6d deficient APP/PS1 mice using immunostaining. We found that plaque associated microglia exhibited substantial accumulation of phosphorylated JAK2 in the processes adjacent to Aβ deposits and soma, and this activation of JAK2 was diminished in Ms4a6d deficient microglia (Fig. 4b and c). Furthermore, we found that Ms4a6d deficient microglia showed pronounced phosphorylated p65 (Fig. 4d and e), an indication of NF-κB activation [52]. These data support our view that Ms4a6d acts as a negative regulator of inflammation in microglia. Interestingly, with immunostaining of phosphorylated p65, we observed elevated signals in Ms4a6d knockout mice following astrocytic processes (Fig. 4f and g), suggesting an up-regulation of inflammatory cytokines released from Ms4a6d deficient microglia.

Activation of NF-κB signaling is associated with subsequent activation of NLRP3 (Nod-like receptor pyrin domain containing 3), which is a critical regulator for inflammasome that controls the release of pro-inflammatory cytokines including IL-1β in AD [53, 54]. We then measured the expression levels of NLRP3 in brain tissues from control and Ms4a6d knockout mice using immunoblotting (Fig. 4h). APP/PS1 mice lacking Ms4a6d showed robust elevation of NLRP3 expression compared to control APP/PS1 mice. (Fig. 4i). Furthermore, we measured the levels of IL-1β using a commercially available ELISA kit and found concurrent increase in IL-1β levels in the brains of Ms4a6d knockout APP/PS1 mice (Fig. 4j).

**Fig. 5:**
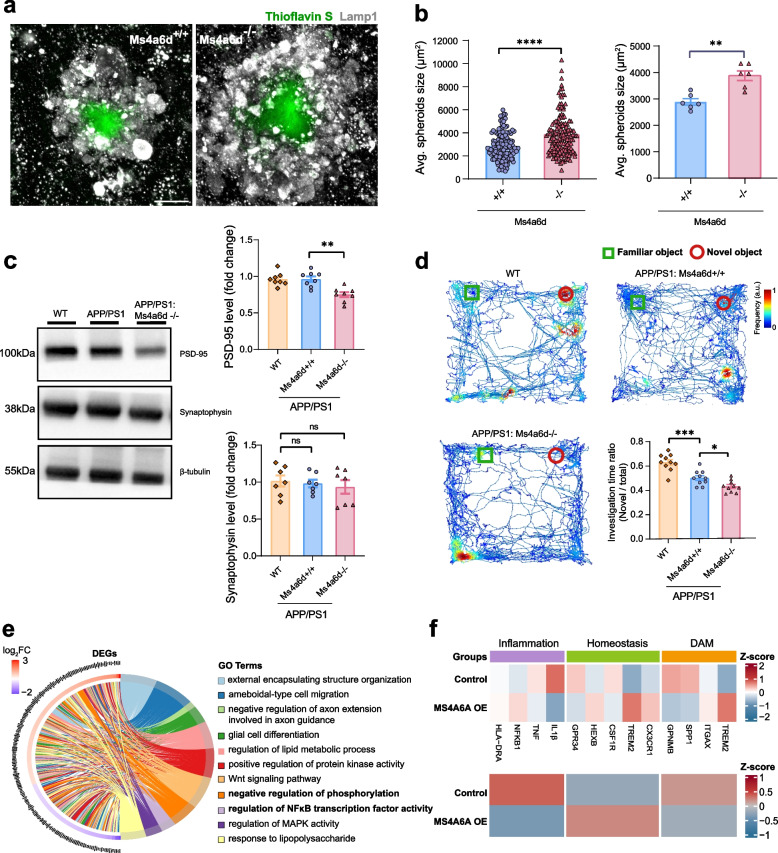
Ms4a6d deficiency exacerbates plaque-associated axonal spheroids, synapse loss and cognitive deficit**. a** Representative images of Lamp1-immunolabeled axonal spheroids (gray) around plaques (green). Scale bar: 10 µm. **b** Quantification of average spheroids size analyzed by individual plaques (left) or by mice (right). Each dot on the right indicates data from individual mouse. *N* = 6 mice for each group. **c** Western blot example images of pre-synaptic marker synaptophysin and post-synaptic marker PSD-95 in hippocampus and the associated quantification. Experiments were replicated for three times. Uncropped gels or western blots can be found at Supplementary Fig. S8. **d** Heatmap of the mice’s trajectories during in novel object recognition tests. The green and red boxes represent the boundaries of the familial and novel object, respectively. *N* = 10 mice for each group. **e** Left semicircular section: differentially expressed genes (DEGs) between MS4A6A over-expression and control groups. (red: log2FC > 0.5; purple: log2FC < −0.5). Right semicircular section: biological pathways with significant differential regulation. **f** Heatmaps of gene sets for inflammation, homeostasis, and disease-associated microglia functions. Color scale represents z-scores of expression levels. Lower panel shows averaged expression from the corresponding gene sets. *N* = 3 repeated experiments. In all panels, data are presented as mean ± S.E.M. Two-tail unpaired Student’s t-test for panel **b**. One-way ANOVA tests with post hoc Tukey tests for panels **d** and **e**. ns, not significant, *, *P* < 0.05, **, *P* < 0.01, ***, *P* < 0.001, ****,* P* < 0.0001. a.u., arbitrary unit

The above results indicated that Ms4a6d deficiency leads to more severe amyloid pathology and microglia-associated neuroinflammation, suggesting that amyloid-induced neurotoxicity may be exacerbated. We next measured the degrees of neurotoxicity with multiple metrics. We have previously established a link between microglia envelopment of plaques and the surrounding axonal spheroids [28, 49], which directly disrupts axonal conduction that is important for neural network functions [33]. Thus, we measured the volume of axonal spheroids around individual amyloid plaques using immunostaining of Lamp1. We found that Ms4a6d deficiency led to an increase in plaque-associated spheroids compared to control group (Fig. 5a and b). In addition, we observed a reduction of postsynaptic marker PSD-95 (Postsynaptic density protein 95) in the brains from Ms4a6d knockout mice, while no change was found in pre-synaptic markers or neuronal cell bodies (Fig. 5c, Supplementary Fig. S4b).

Finally, we conducted behavioral assessments on Ms4a6d-knockout mice. Utilizing the novel object recognition test, we mapped the trajectories of the mice in an open field containing both familiar and novel objects. The heat maps provided a visualization of the changes in the mice's positions, with red indicating longer durations spent in specific areas. Our findings revealed that, compared to age-matched APP/PS1 mice, the Ms4a6d-knockout mice exhibited a decreased proportion of their total exploration time on the novel object (Fig. 5d). This lack of preference for the novel object suggests more severe cognitive impairment in the Ms4a6d-knockout mice.

The above results indicated that MS4A6A/Ms4a6d deficiency could disrupt microglia response to amyloid plaques thus exacerbating the risks of developing AD. It is thus possible that enhancing *MS4A6A* expression could provide a protective effect. To explore this possibility, we performed *MS4A6A* overexpression in the HMC3 cell line. RNA sequencing of *MS4A6A* overexpressed cells revealed 851 DEGs (FDR < 0.1, |log2FC|> 0.5) (Supplementary Fig. S5, Supplementary Material 2). *MS4A6A* overexpression exerted a broad range of gene expression regulation for microglia including inflammatory signaling pathways (e.g., NF-κB and response to LPS), glial activation, protein kinase activity regulation (regulation of phosphorylation), phagocytosis-related pathways (encapsulating), and migration-associated modules (Fig. 5e). Consistent with our results, we find the general effect of *MS4A6A* to be anti-inflammation and at the same time activating plaque-responsive genes such as *TREM2* (Fig. 5f) [55]. This is consistent with prior reports of coordinated MS4A-TREM2 upregulation in protective microglial states [12], supporting the potential protective effect of MS4A6A upregulation.

Altogether, these data indicated that Ms4a6d deficiency disrupts microglia phagocytosis and envelopment of amyloid, and exacerbates neuroinflammation and neurotoxicity.

## Discussion

Although the link between *MS4A6A* mutations and AD has been previously identified [5, 8, 44], the cellular mechanism underlying the involvement of this gene and AD pathogenesis is not known. A prior study identified increased levels of plasma MS4A6A in human AD patients and argued that MS4A6A inhibition could be therapeutically beneficial [19]. Utilizing a knockout model of the homologue gene *Ms4a6d* in mice, we found that Ms4a6d is a master switch controlling microglia response to amyloid pathology. Lacking Ms4a6d greatly diminishes microglia clearance of amyloid via phagocytosis, and the neuroprotective barrier function that insulates and compresses plaques. At the same time, Ms4a6d deficiency exacerbates plaque-associated inflammatory responses in microglia. All these phenotypes point to a congruent effect that Ms4a6d deficiency promotes the pathogenesis of AD, thus it is likely that the upregulation seen with aging and in AD patients are compensatory mechanisms. Interestingly, the disruption of microglia barrier and exacerbated axonal spheroids we observed with Ms4a6d knockout has been a recurring theme related to AD genetic risks, including *TREM2 R47H* and *APOE4* [28, 31, 56]. These data suggest that pathologies such as axonal spheroids could be a common mechanism for AD pathogenesis [33]. Previous GWAS studies reported several protective mutations of *MS4A6A*, and our results reported novel risk alleles. All these loci locate on the non-coding sequences. Thus, they likely modulate AD risks by changing the expression level of *MS4A6A* as we showed for rs646924. While our current study indicates that MS4A6A deficiency disrupts microglial protective function and enhances inflammation, future studies are required to elucidate the precise mechanisms correspond to individual mutations on the *MS4A6A* gene.

We propose that enhancing MS4A6A function could be an ideal therapeutic target for modulating microglia to treat AD (Supplementary Fig. S6). Despite ample evidence with human genetics and mouse models, no microglia-specific target is currently available for AD treatment. Previous clinical trials aiming to suppress neuroinflammation as AD treatment did not yield positive results [57–59], indicating that broad-spectrum inhibition of microglial activity may not be an effective target. Furthermore, several studies have attempted to deplete inflammatory microglia with pharmacological antagonists of colony-stimulating factor-1 receptor [60]. This approach effectively depletes microglia in the brain, but the results on AD pathology are mixed. While reduced plaque depositions and improved cognition have been reported in some studies, microglia depletion can lead to acceleration of plaque growth [61], more diffuse amyloid structures [62–66], and exacerbated axonal spheroids as well as cerebral amyloid angiopathy [67–69].

Additionally, current passive anti-Aβ immunization approach also alters microglial function [70]. Antibodies against Aβ-peptide attract microglia to amyloid pathologies via the Fc fragment to boost clearance [71–73] and microglia barrier [49]. But activation of the Fc receptor in microglia also induces heightened inflammation [74–76], which could contribute to neuronal damages associated with this treatment [77]. Another treatment approach that specifically engages microglia is the recently developed activation antibody for TREM2 [78–80]. These antibodies act by promoting the interaction between TREM2 and the downstream DAP12 (DNAX-activating protein of 12 kDa) adapter, leading to increased microglial encapsulation and phagocytosis of amyloid deposits [79, 81, 82]. However, some studies also reported that TREM2 activation enhances neuroinflammation [83–86]. Therefore, effective intervention should ideally have differential effect on the divergent functions of microglia, yet currently such approach is not available.

Other potential targets for modulating microglia include NLRP3 [53] and HK2 (Hexokinase 2) [26]. NLRP3 is crucial for inflammasome formation. And NLRP3 knockout reduces neuroinflammation and increase phagocytosis, yet it does not seem to affect microglial encapsulation of amyloid deposits [54]. HK2 is an essential enzyme for glycolysis. And HK2 knockout alters microglial metabolism, enhancing phagocytosis but also increasing inflammation [87]. In contrast, we found that Ms4a6d knockout in mice inhibits microglial encapsulation and phagocytosis and increases inflammation. Thus, it is possible that boosting MS4A6A in microglia would be beneficial for both aspects. Interestingly, a recent analysis of MS4A4A polymorphism revealed similar phenotypes of suppressed inflammation and enhanced phagocytosis, despite low similarity in their protein sequences [88]. Future studies are required to elucidate the distinct and related functions of these genes.

This study had several limitations. Firstly, although the mouse homologue Ms4a6d shows the highest similarity, *MS4A6A* generates multiple isoforms that likely serve different functions. Future studies are required to dissect this diversity in details. Secondly, this study illustrated that Ms4a6d deficiency regulated both microglia functions, while it remains to be determined whether Ms4a6d overexpression could be beneficial in vivo. Thirdly, high quality antibody for Ms4a6d would greatly facilitate the functional study of this gene. Future research identifying effective ways of activating MS4A6A could potentially constitute a novel approach for microglia modulation for treating AD.

## Conclusions

Our study reported several novel genetic polymorphisms on human *MS4A6A* gene that are associated with altered risks for AD onset and human Aβ level in CSF. MS4A6A deficiency in microglia blocks neuroprotection and worsens neurotoxicity which is a key modulator for AD pathogenesis. We pointed out an exciting possibility that boosting MS4A6A could achieve precise regulations of microglia function that are beneficial in both aspects. Future development of feasible approaches on this target could likely be effective in preventing the development of AD.

## Supplementary Information

Supplementary Material 1.

Supplementary Material 2.

## Data Availability

All data needed to evaluate the conclusions in the paper are present in the paper and/or the Supplementary Materials.

## Abbreviations

AD: Alzheimer’s disease
SNP: Single nucleotide polymorphism
MS4A6A: Membrane spanning 4-domains A 6A
TREM2: Triggering receptor expressed on myeloid cells 2
MS4A: Membrane spanning 4 A
Aβ: β-Amyloid
CABLE: Chinese Alzheimer’s Biomarker and LifestylE
MoCA: Montreal Cognitive Assessment
MCI: Mild cognitive impairment
CSF: Cerebrospinal fluid
ISF: Interstitial fluid
ELISA: Enzyme-linked immunosorbent assay
GWAS: Genome-wide association studies
O.C.T.: Optimal cutting temperature
Stereo-seq: SpaTial Enhanced REsolution Omics sequencing
PBS: Phosphate-buffered saline
Iba1: Ionized calcium binding adapter molecule 1
Lamp1: Lysosome associated membrane protein 1
JAK2: Janus kinases 2
IL-1β: Interleukin-1β
S.E.M.: Standard Error of Mean
NF-κB: Nuclear factor κB
NLRP3: Nod-like receptor pyrin domain containing 3
PSD95: Postsynaptic density protein 95
DEG: Differentially expressed genes
HK2: Hexokinase 2
