# Supplementary material for: MS4A6A/Ms4a6d deficiency disrupts neuroprotective microglia functions and promotes inflammation in Alzheimer’s disease model
Source: Mol Neurodegener. 2025 Aug 28;20:94. doi: 10.1186/s13024-025-00887-0 (PMC12395693; doi:10.1186/s13024-025-00887-0)
Supplement: Supplementary file 1 — Supplementary Material 1. [file 13024_2025_887_MOESM1_ESM.docx]

### Supplementary Information

[Supplementary Information 1](#_Toc202187777)

[Supplementary Fig. S1 Linkage disequilibrium (LD) patterns among SNPs 2](#_Toc202187778)

[Supplementary Fig. S2 Structure homology analysis between MS4A6A and Ms4a6d 3](#_Toc202187779)

[Supplementary Fig. S3 Ms4a6d knockout mouse model generation strategy and validation 4](#_Toc202187780)

[Supplementary Fig. S4 Microglia and neuron number in APP/PS1 mice with different Ms4a6d genotypes 6](#_Toc202187781)

[Supplementary Fig. S5 Volcano map of differentially expressed genes 7](#_Toc202187782)

[Supplementary Fig. S6 Graphic abstract 8](#_Toc202187783)

[Supplementary Fig. S7 Uncropped western blot source data for Figure 4 9](#_Toc202187784)

[Supplementary Fig. S8 Uncropped western blot source data for Figure 5 10](#_Toc202187785)

[Supplementary Table S1. Demographic characteristics of CABLE study 11](#_Toc202187786)

[Supplementary Table S2. Homology analysis between MS4A6A and Ms4a6d using BLAST 13](#_Toc202187787)

### Supplementary Fig. S1 Linkage disequilibrium (LD) patterns among SNPs


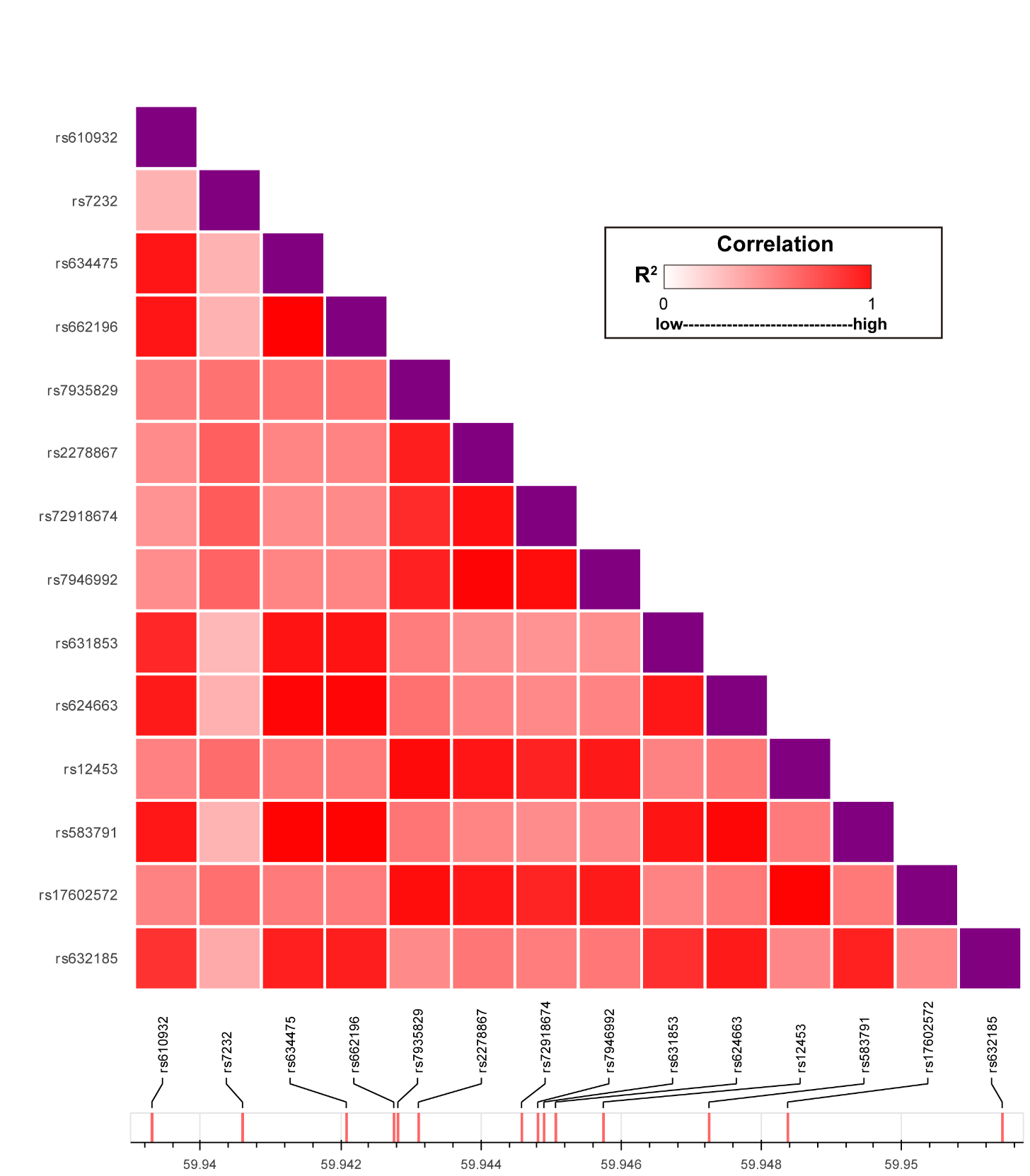


### Supplementary Fig. S2 Structure homology analysis between MS4A6A and Ms4a6d


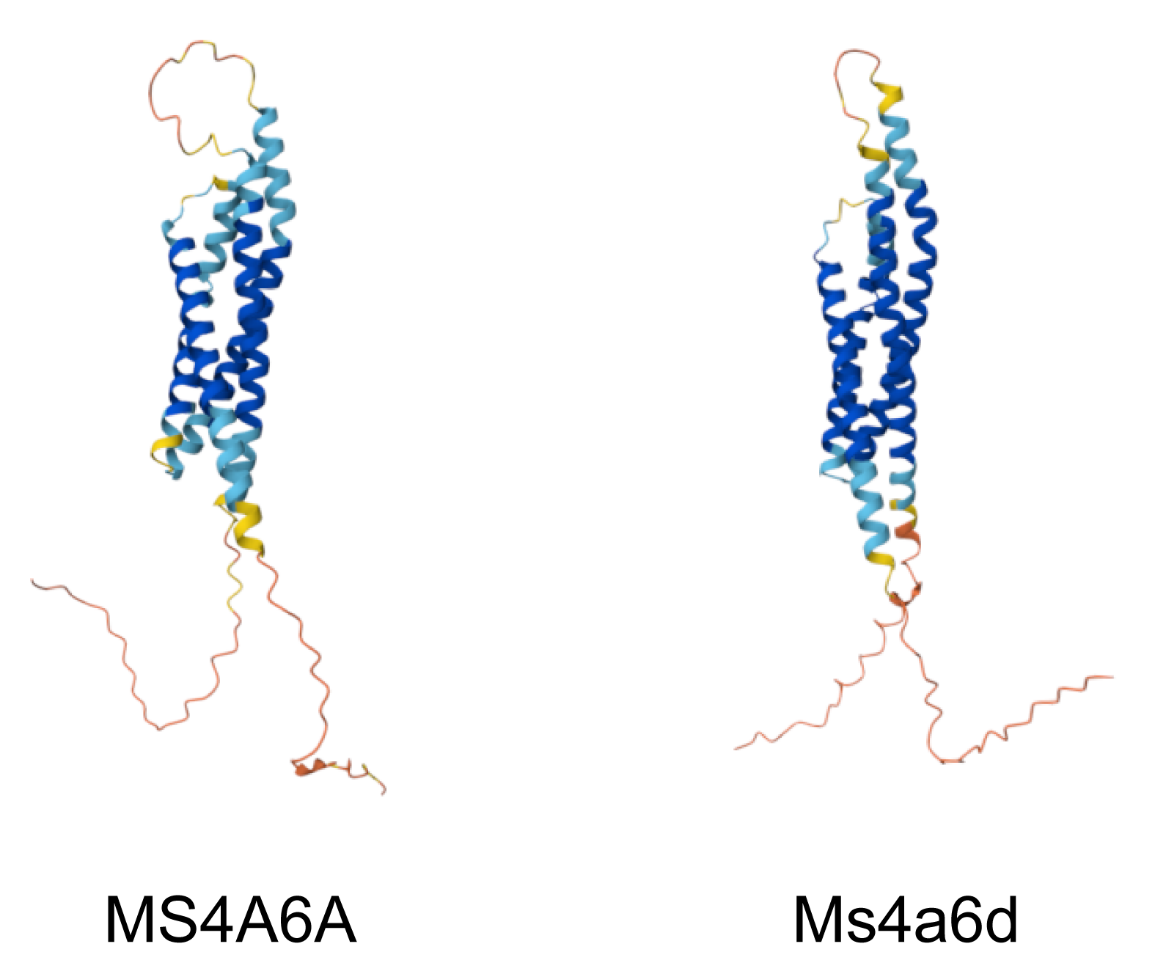


### Supplementary Fig. S3 Ms4a6d knockout mouse model generation strategy and validation


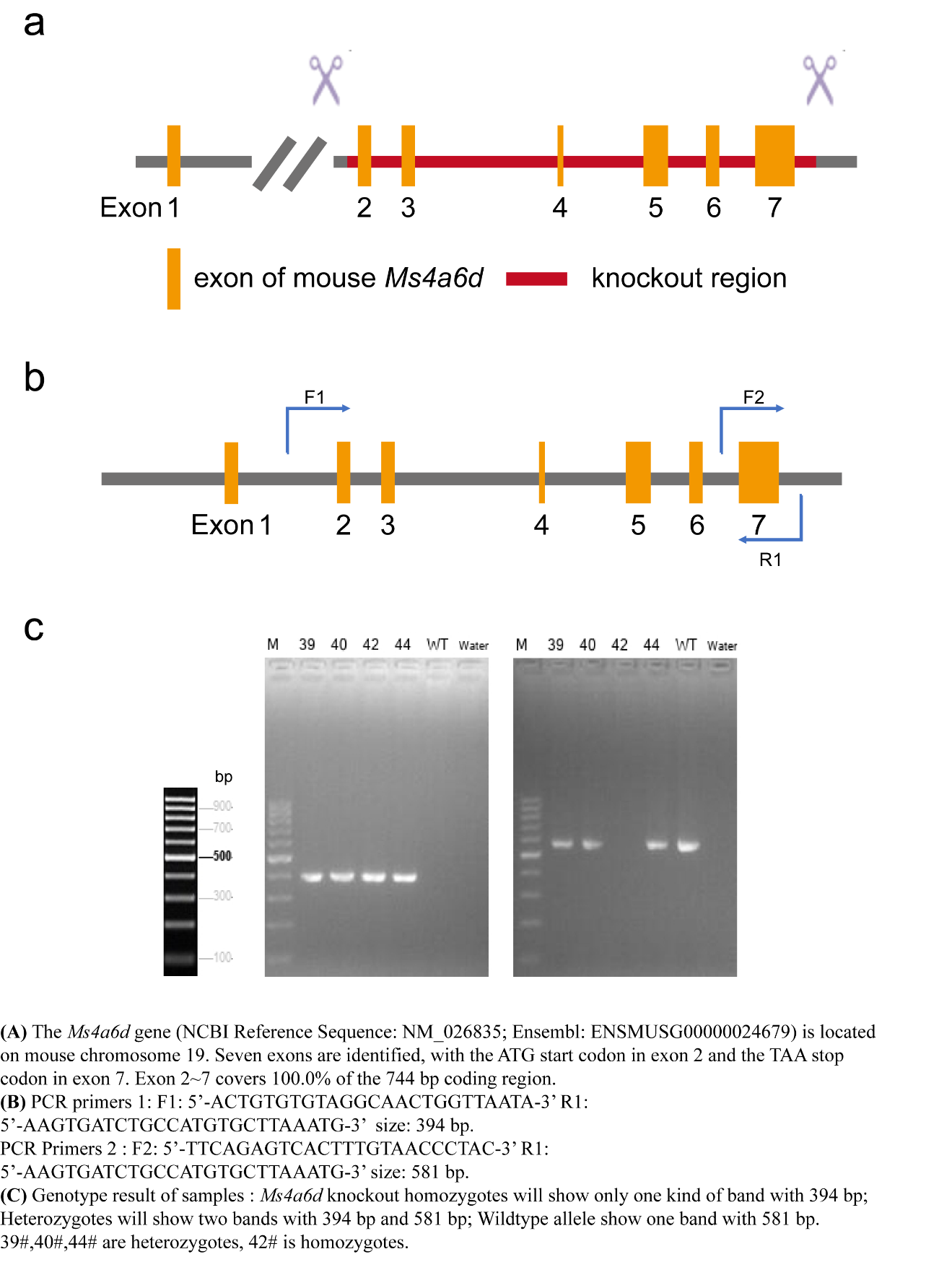


(a) The Ms4a6d gene (NCBI Reference Sequence: NM_026835; Ensembl: ENSMUSG00000024679) is located on mouse chromosome 19. Seven exons are identified, with the ATG start codon in exon 2 and the TAA stop codon in exon 7. Exon 2~7 covers 100.0% of the 744 bp coding region.

(b) PCR primers 1: F1: 5’-ACTGTGTGTAGGCAACTGGTTAATA-3’ R1: 5’-AAGTGATCTGCCATGTGCTTAAATG-3’ size: 394 bp.

PCR Primers 2: F2: 5’-TTCAGAGTCACTTTGTAACCCTAC-3’ R1: 5’-AAGTGATCTGCCATGTGCTTAAATG-3’ size: 581 bp.

(c) Genotype result of samples: Ms4a6d knockout homozygotes will show only one kind of band with 394 bp; Heterozygotes will show two bands with 394 bp and 581 bp; Wildtype allele show one band with 581 bp. 39#,40#,44# are heterozygotes, 42# is homozygotes.

### Supplementary Fig. S4 Microglia and neuron number in APP/PS1 mice with different Ms4a6d genotypes


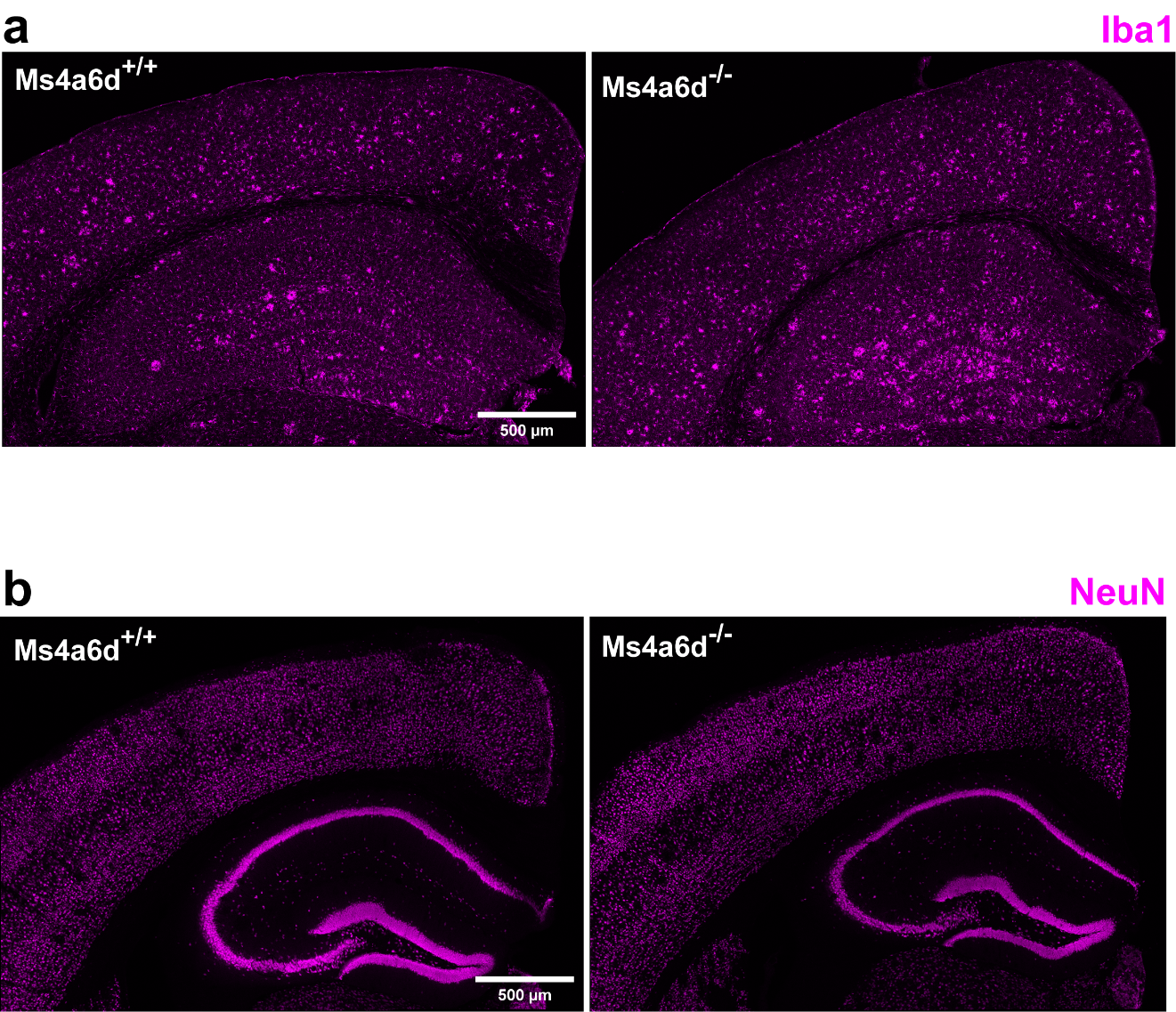


Ms4a6d deletion did not affect the overall distribution and density of (a) microglia and (b) neuron

### Supplementary Fig. S5 Volcano map of differentially expressed genes


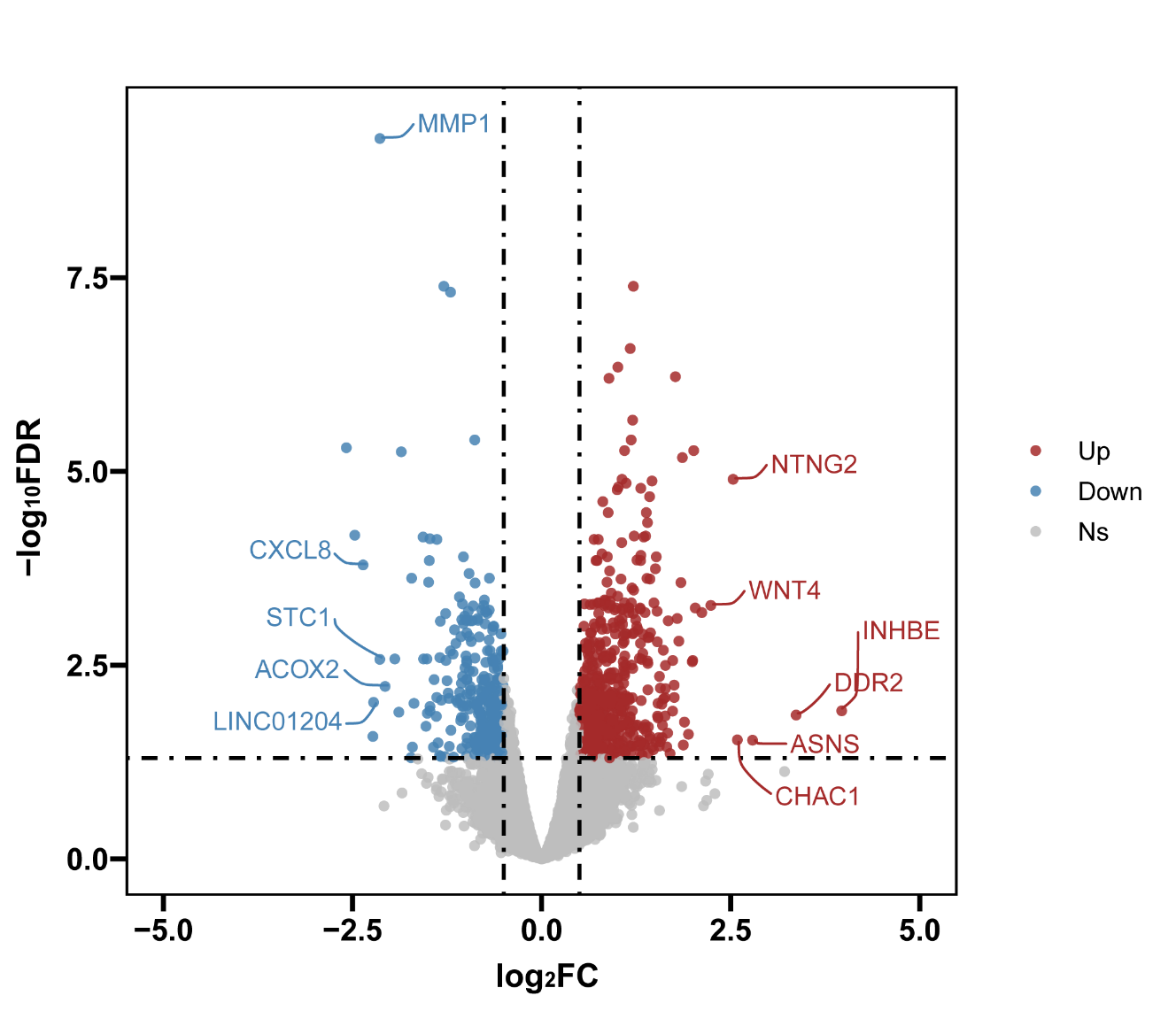


### Supplementary Fig. S6 Graphic abstract


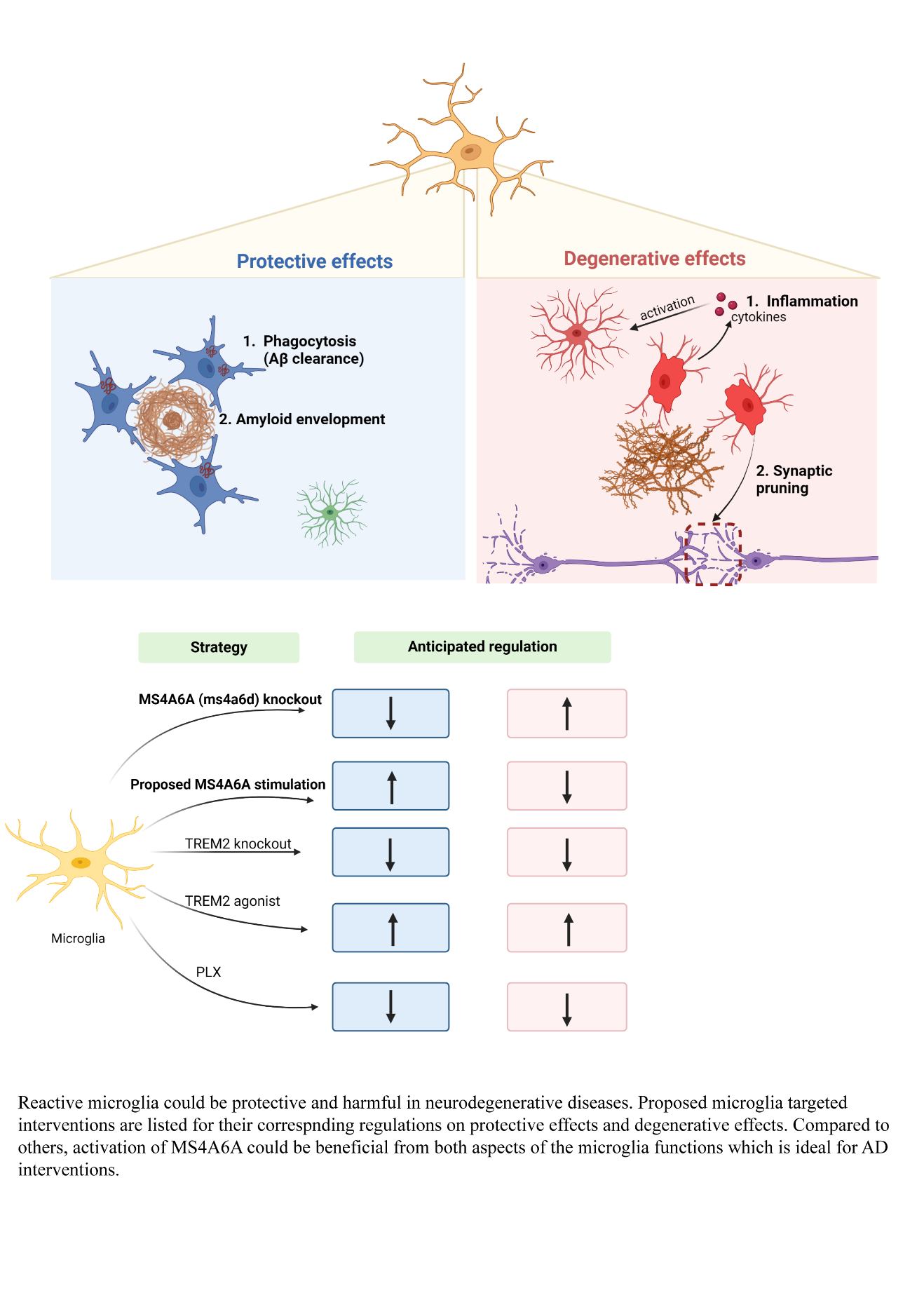


Reactive microglia could be protective and harmful in neurodegenerative diseases. Proposed microglia targeted interventions are listed for their corresponding regulations on protective effects and degenerative effects. Compared to others, activation of MS4A6A could be beneficial from both aspects of the microglia functions which is ideal for AD interventions

### Supplementary Fig. S7 Uncropped western blot source data for Figure 4


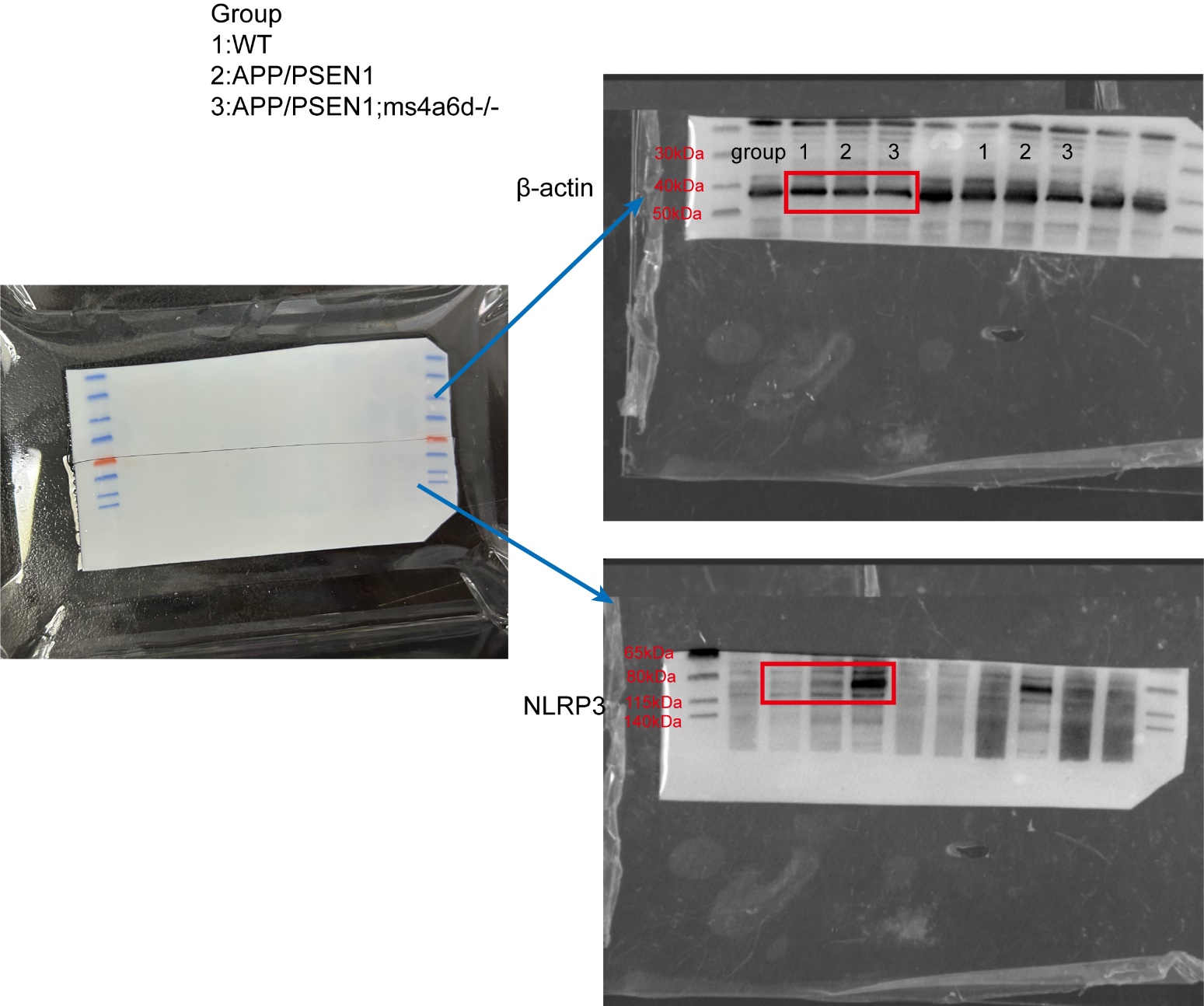


### Supplementary Fig. S8 Uncropped western blot source data for Figure 5


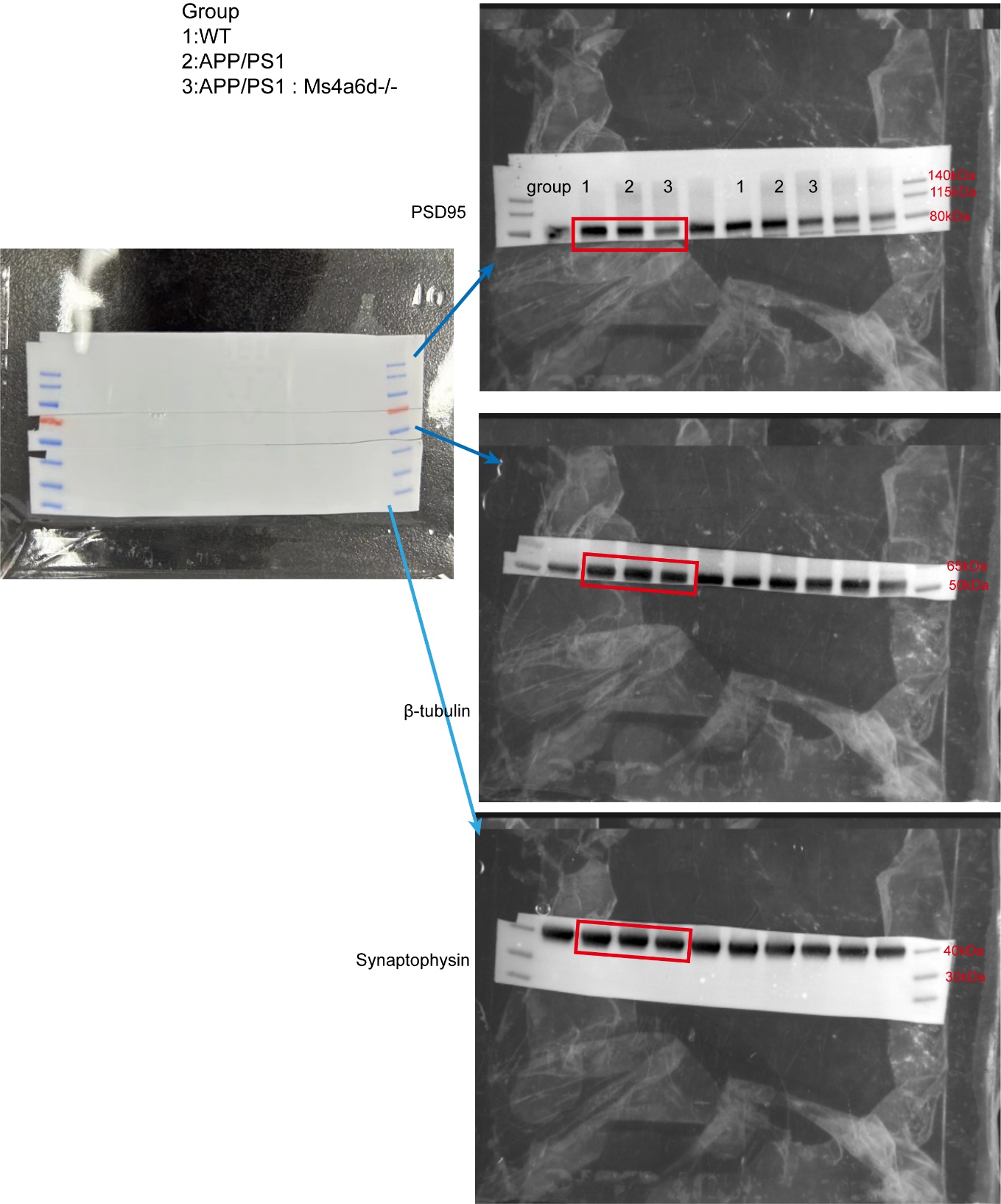


### Supplementary Table S1. Demographic characteristics of CABLE study

| **Characteristics** | **CABLE study** | | | |
| --- | --- | --- | --- | --- |
|  | **Normal cognition** | **Mild cognition impairment** | **Total** | ***P*** |
|  | (N=537) | (N=453) | (N=990) |  |
| **Age (years)** | 59.2 (± 10.0) | 64.7 (± 9.17) | 61.7 (± 10.0) | <0.001 |
| **Sex** |  |  |  |  |
| Female | 234 (43.6%) | 176 (38.9%) | 410 (41.4%) | 0.323 |
| Male | 303 (56.4%) | 277 (61.1%) | 580 (58.6%) |  |
| **Education (years)** | 9.85 (± 4.58) | 9.83 (± 3.18) | 9.84 (± 4.00) | 0.801 |
| **APOE ε4 status** |  |  |  | 0.140 |
| ε4-/- | 471 (87.7%) | 381 (84.1%) | 852 (86.1%) |  |
| ε4+/- | 65 (12.1%) | 65 (14.3%) | 130 (13.1%) |  |
| ε4+/+ | 1 (0.2%) | 7 (1.5%) | 8 (0.8%) |  |
| **MMSE** | 28.5 (± 1.65) | 27.2 (± 1.98) | 27.9 (± 1.92) | <0.001 |
| **MOCA** | 25.9 (± 2.74) | 20.3 (± 3.34) | 23.3 (± 4.12) | <0.001 |
| **CSF Aβ42 (pg/ml)** | 376 (± 223) | 396 (± 255) | 385 (± 238) | 0.663 |
| **CSF Aβ40 (pg/ml)** | 6510 (± 3580) | 6810 (± 3450) | 6650 (± 3520) | 0.224 |
| **CSF Aβ42/Aβ40** | 0.0657 (± 0.0584) | 0.0643 (± 0.0502) | 0.0651 (± 0.0548) | 0.956 |
| **CSF p-tau181 (pg/ml)** | 42.1 (± 13.8) | 46.5 (± 16.1) | 44.1 (± 15.0) | <0.001 |
| **CSF t-tau (pg/ml)** | 188 (± 90.4) | 220 (± 115) | 203 (± 104) | <0.001 |
| **CSF sTREM2 (pg/ml)** | 16900 (± 7640) | 18500 (± 6660) | 17600 (± 7250) | 0.005 |
| **CSF progranulin (pg/ml)** | 1730 (± 262) | 1730 (± 243) | 1730 (± 253) | 0.575 |
| **CSF α-synuclein (pg/ml)** | 1200 (± 779) | 1430 (± 1530) | 1310 (± 1180) | 0.0912 |

Data were shown as mean (standard deviations, SD) for continuous variables, and was show as number (percentage) for categorical variables. P values were analyzed using Student's t test for continuous variables and the Chi-square test for categorical variables

### Supplementary Table S2. Homology analysis between MS4A6A and Ms4a6d using BLAST

| **Protein** | **E value** | **Percent identity** | **UniprotKB** |
| --- | --- | --- | --- |
| Membrane-spanning 4-domains subfamily A member 6D | 2e-69 | 55.30% | Q99N07.1 |
| Membrane-spanning 4-domains subfamily A member 6D | 6e-69 | 54.84% | NP_081111.1 |
| Membrane-spanning 4-domains subfamily A member 6B | 5e-68 | 53.92% | NP_081485.2 |
| Membrane-spanning 4-domains subfamily A member 6C isoform 1 | 4e-57 | 50.47% | NP_082871.2 |

Homology analysis between MS4A6A and other proteins, showing that Ms4a6d having the highest similarity
